# Transposable element-assisted evolution and adaptation to host plant within the *Leptosphaeria maculans*-*Leptosphaeria biglobosa* species complex of fungal pathogens

**DOI:** 10.1186/1471-2164-15-891

**Published:** 2014-10-12

**Authors:** Jonathan Grandaubert, Rohan GT Lowe, Jessica L Soyer, Conrad L Schoch, Angela P Van de Wouw, Isabelle Fudal, Barbara Robbertse, Nicolas Lapalu, Matthew G Links, Bénédicte Ollivier, Juliette Linglin, Valérie Barbe, Sophie Mangenot, Corinne Cruaud, Hossein Borhan, Barbara J Howlett, Marie-Hélène Balesdent, Thierry Rouxel

**Affiliations:** INRA-Bioger, UR1290, Avenue Lucien Brétignières, BP 01, 78850 Thiverval-Grignon, France; School of Botany, The University of Melbourne, Melbourne, Victoria 3010 Australia; NIH/NLM/NCBI, 45 Center Drive, MSC 6510, Bethesda, Maryland 20892-6510 USA; INRA-Bioger, URGI Route de St Cyr, 78026 Versailles Cedex, France; Agriculture and Agri-Food Canada/Agriculture et Agroalimentaire Canada, Saskatoon Research Centre, 107 Science Place, Saskatoon, SK S7N OX2 Canada; Department of Computer, University of Saskatchewan, 110 Science Place, Saskatoon, SK S7N 5C9 Canada; GENOSCOPE, Centre National de Séquençage, Institut de Génomique CEA/DSV, 2, rue Gaston Crémieux, CP 5706, 91057 Evry Cedex, France

**Keywords:** Comparative genomics, Fungal plant pathogen, Transposable elements, Effector genes, Speciation, Adaptation to host

## Abstract

**Background:**

Many plant-pathogenic fungi have a tendency towards genome size expansion, mostly driven by increasing content of transposable elements (TEs). Through comparative and evolutionary genomics, five members of the *Leptosphaeria maculans*-*Leptosphaeria biglobosa* species complex (class *Dothideomycetes*, order *Pleosporales*), having different host ranges and pathogenic abilities towards cruciferous plants, were studied to infer the role of TEs on genome shaping, speciation, and on the rise of better adapted pathogens.

**Results:**

*L. maculans* ‘brassicae’, the most damaging species on oilseed rape, is the only member of the species complex to have a TE-invaded genome (32.5%) compared to the other members genomes (<4%). These TEs had an impact at the structural level by creating large TE-rich regions and are suspected to have been instrumental in chromosomal rearrangements possibly leading to speciation. TEs, associated with species-specific genes involved in disease process, also possibly had an incidence on evolution of pathogenicity by promoting translocations of effector genes to highly dynamic regions and thus tuning the regulation of effector gene expression *in planta*.

**Conclusions:**

Invasion of *L. maculans* ‘brassicae’ genome by TEs followed by bursts of TE activity allowed this species to evolve and to better adapt to its host, making this genome species a peculiarity within its own species complex as well as in the *Pleosporales* lineage.

**Electronic supplementary material:**

The online version of this article (doi:10.1186/1471-2164-15-891) contains supplementary material, which is available to authorized users.

## Background

Fungi (and fungal-like oomycetes) are an incredibly diverse and adaptable group of organisms which colonise all habitats on Earth. Some are plant and animal pathogens of major economic importance and as stressed by Kupferschmidt [1], “Fungi have now become a greater global threat to crops, forests, and wild animals than ever before. They have killed countless amphibians, pushing some species to extinction, and they are threatening the food supply for billions of people.” The sequencing of fungal genomes (the yeast *Saccharomyces cerevisiae* [2], followed by filamentous ascomycetes such as *Neurospora crassa* [3], and later plant pathogenic fungi such as *Ustilago maydis*, *Magnaporthe oryzae*, *Fusarium graminearum* and *Phaeosphaeria nodorum* [4–7]) initially indicated that genomes of filamentous ascomycetes are relatively small (21–39 Mb), with few repetitive elements (typically less than 10%) and they contain 10,000-15,000 protein-encoding genes. Unlike symbiotic and parasitic bacteria, which usually have smaller genomes than their free-living relatives [8], the first genome data from filamentous fungi did not indicate differences between genome size of fungal pathogens and that of saprophytes. However, contrasting to what is observed in bacteria, recent sequence data indicate that some phytopathogens have drastically expanded genomes, mostly due to massive invasion by Transposable Elements (TEs).

The 45-Mb genome of *Leptosphaeria maculans*, the ascomycete that causes stem canker of crucifers contains 33% TEs, the 120-Mb genome of cereal downy mildew, *Blumeria graminis*, contains 64% TEs (while other closely related downy mildew pathogens have even larger genomes), and that of the 240-Mb oomycete *Phytophthora infestans* contains 74% TEs [8]. These data, suggesting convergent evolution towards bigger genomes in widely divergent fungal and oomycete species, are intriguing and the counterbalance between selective advantage conferred to the pathogen and the cost of maintaining this amount of “parasitic” DNA is unknown.

Analysis of *L. maculans* ‘brassicae’ (Lmb) genome [9] revealed a genomic structure at this time only observed in mammals and other vertebrates: the base composition (GC-content) varied widely along the chromosomes, but locally, was relatively homogeneous. Such structural features of chromosomes are termed “isochores” [10]. In Lmb, the high TE proportion in conjunction with RIP (Repeat-Induced Point mutation) [11] generates large AT-rich regions (33.9% GC-content), called AT-isochores. These AT-isochores are scattered along the genome alternating with large GC-equilibrated regions (51% GC-content), GC-isochores, containing 95% of the predicted genes and mostly devoid of TEs. AT-isochores cover 36% of *L. maculans* genome, are mainly composed of mosaics of truncated and RIP-degenerated TEs and only contain 5% of the predicted genes, but 20% of those genes encode small secreted proteins (SSPs) considered as putative effectors of pathogenicity [9]. As only 4% of the genes located in GC-isochores encode SSPs, we hypothesized that AT-isochores were niches for putative effectors. This was corroborated by the presence within these AT-isochores of genes encoding known effectors, including four avirulence genes (*i.e*. encoding proteins recognised by plant resistance genes), *AvrLm1* [12], *AvrLm6* [13], *AvrLm4*-*7* [14] and *AvrLm11* [15]. Moreover, genes within AT-isochores are affected by RIP, which can occasionally overrun the repeated region into adjacent single-copy genes, resulting in extensive mutation of the affected genes [16–18]. This genome environment favors a rapid response to selection pressure. For example, effector genes acting as avirulence genes can be inactivated in a single sexual cycle due to RIP or deletions due to large-scale genome rearrangements in AT-isochores [16, 18, 19].

In addition to genes encoding effectors and genes with no predicted function, AT-isochores are also enriched in gene clusters responsible for the biosynthesis of secondary metabolites [9]. Both effector genes and secondary metabolite gene clusters are distributed discontinuously among *ascomycetes* and/or show species specificity [9, 20]. We have postulated that genome invasion by TEs, followed by their degeneracy by RIP shaping large AT-isochores, is a recent evolutionary event that has contributed to the rise of a better adapted new species, and that maintaining large TE-rich regions hosting pathogenicity determinants has favoured adaptation to new host plants along with generation of new virulence specificities [9].

This hypothesis can be now tested by exploiting comparative genomics between Lmb and other members of the *Leptosphaeria* species complex, for which preliminary electrokaryotype and Southern blot analyses suggested only limited TE content (Additional file 1: Figure S1). *L. maculans* and *L. biglobosa* are dothideomycete phytopathogens specialized on crucifers and encompassing a series of ill-defined entities more or less adapted to oilseed rape/canola (*Brassica napus*). Some of the species of the complex attack oilseed rape and are responsible for the main disease of the crop (blackleg or Phoma stem canker) [21–23]. The complex also encompasses related lineages only found on cruciferous weeds. These include *L. maculans* ‘lepidii’ (Lml) isolated from *Lepidium* spp. and *L. biglobosa* ‘thlaspii’ (Lbt) isolated from *Thlaspi arvense*, but also found occasionally on *Brassica* species in Canada [21, 22]. The *L. maculans* and *L. biglobosa* lineages infecting *B. napus* (Lmb, *L. biglobosa* ‘brassicae’ (Lbb) and *L. biglobosa* ‘canadensis’ (Lbc)) share morphological traits, epidemiology, initial infection strategies, ecological niches and regardless of geographic distribution, are often found together in tissues of individual infected plants [24].

In this paper we investigate when and how genome expansion took place in the *L. maculans*-*L. biglobosa* species complex, and the consequences it had on genome structure, adaptability and pathogenicity. The genomes of five members of the species complex (Lml, Lbb, Lbc, Lbt and another isolate of Lmb) were sequenced. The genomic data were analysed and compared to those from other dothideomycete species to address the following questions: (i) Do the different members of the species complex constitute separate biological species and did TEs contribute to speciation? (ii) Did TE invasion contribute to the rise of a better adapted and more adaptable species: *i.e*. what was the incidence of TE invasion on the generation, modification, loss or regulation of pathogenicity determinants?

## Results

### Phylogeny and estimates of divergence times

**Figure 1:**
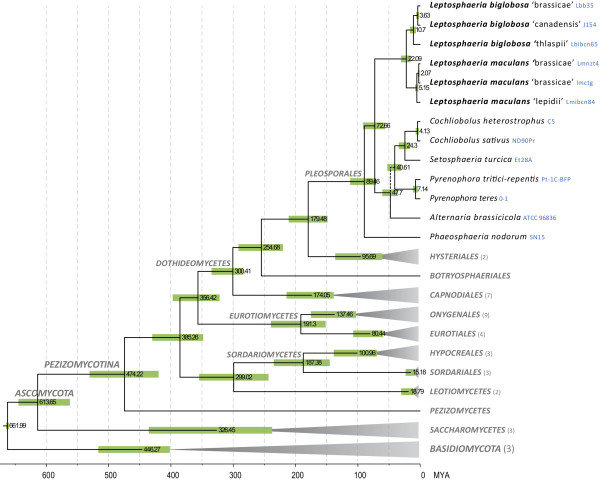
**Chronogram of major classes in** ***Ascomycota***, **with a focus on** ***Dothideomycetes.*** The chronogram was produced with BEAST from a data set of 19 truncated proteins. Branches with terminal grey triangles were collapsed and their number of leaves indicated in brackets after taxon labels. Numbers at nodes indicate mean node ages in millions of years and green bars indicate their 95% highest posterior density intervals. A broken line indicates uncertain phylogenetic placement.

*L. maculans* and *L. biglobosa* belong to order *Pleosporales* from the class *Dothideomycetes*. This class is the largest and the most phylogenetically diverse within *Pezizomycotina* (filamentous ascomycetes), and includes numerous plant pathogens such as species in the genera *Cochliobolus*, *Phaeosphaeria*, *Pyrenophora*, *Mycosphaerella*, *Zymoseptoria* and *Venturia* [25, 26] (Figure 1).

The chronogram presented in Figure 1 (for a more detailed analysis see Additional file 2: Figure S2), based on the alignment of 19 conserved proteins, has representatives from three of the four main classes in the *Pezizomycotina*, but the main focus is on *Dothideomycetes*. This phylogeny is congruent with other more complete analyses [25, 27] and indicates that the *Leptosphaeria* species diverged from the sampled plant pathogens of Pleosporaceae (*Cochliobolus*, *Pyrenophora* and *Alternaria*) approximately 73 MYA.

Based on morphological features, *L. maculans* and *L. biglobosa* were considered as two pathotypes of the same species for more than 70 years [28, 29] until formal renaming in 2001 [30]. Our data estimate that *L. maculans* and *L. biglobosa* diverged *ca*. 22 MYA (Figure 1).

Divergence times were also determined within the *L. maculans* and *L. biglobosa* lineages. Voigt *et al*. [22] suggested that Lbt was distinct from the other *L. biglobosa* sub-clades, which is supported by our current data since it diverged *ca*. 11 MYA from the terminal Lbb or Lbc (Figure 1). In terminal branches, divergence time between Lmb and Lml (5.1 MYA) and that of Lbb and Lbc (3.6 MYA) are comparable (Figure 1).

### Genome statistics

The reference genome of Lmb (of isolate v23.1.3), was sequenced in 2007 using a whole genome shotgun strategy and Sanger technology [9]. Here, Next Generation Sequencing (NGS) technologies were used to sequence the genomes of five members of the species complex, including another isolate of Lmb. Despite different sequencing and assembly strategies, the resequenced isolate of Lmb (WA74) had a similar genome size and proportion of TEs (25.8%) compared to v23.1.3 (32.5% of TEs) (Table 1).

**Table 1:** **Sequencing statistics and genome statistics for six members of the** ***Leptosphaeria maculans***-***L. biglobosa*** **species complex**

	***L. maculans***‘brassicae’		***L. maculans***‘lepidii’	***L. biglobosa***‘brassicae’	***L. biglobosa***‘canadensis’	***L. biglobosa***‘thlaspii’
	v23.1.3	WA74	IBCN84	B3.5	J154	IBCN65
Genome size (Mb)	45.1	44.2	31.5	31.8	30.2	32.1
Contig number	1743	3765	2802	2533	7124	3506
Scaffold number	76	986	123	606	6748	237
Scaffold N50 (kb)	1770	263	1356	779	245	715
Gaps (%)	2.5	9.6	7.1	7.4	0.1	8.7
Repeats (%)	35.5	27.5	4.0	4.4	3.9	5.1
Transposable elements (TEs) (%)	32.5	25.8	2.7	3.2	2.9	4.0
‘Ancestral’ genome size (Mb)^a^	29.3	28.5	28.4	28.4	29.3	28.0
GC content of the whole genome (%)	45.2	46.5	50.9	51.4	51.1	51.4
GC ‘ancestral’ genome’ (%)	51.6	51.6	51.6	52.0	51.6	52.1
GC content of TEs (%)	34.3	34.5	32.3	36.6	34.7	36.6
Predicted gene number	12543	10624	11272	11390	11068	11691

^a^‘Ancestral genome’ refers to the core genome without TEs.

Compared to the Lmb isolates, the other members of the species complex had smaller genomes, ranging from 30.2 to 32.1 Mb and only comprising 2.7% to 4.0% of TEs. These sequencing data are consistent with electrokaryotypes done using Pulsed Field Gel Electrophoresis (PFGE) and results of DNA hybridization with known TEs (Additional files 1 and 3: Figures S1, S5).

The GC-content of the Lmb reference genome was 45%, which is similar to that of Lmb isolate WA74 (Table 1). In contrast, all other isolates of the species complex had genome GC-contents (*ca*. 51%, Table 1) that related more to those of other *Dothideomycetes* (50-52%, Additional file 4: Table S1), with a couple of exceptions discussed below [26, 31, 32]. These data indicate that Lmb is an exception in terms of TE-content compared to other *Pleosporales* including closely related members of the species complex (Figure 1). Only dothideomycete species of order *Capnodiales* such as *Pseudocercospora fijiensis* and *Cladosporium fulvum* have genomes enriched in TEs, resulting in low overall GC-content of their genome [26, 31] (Additional file 4: Table S1).

In the *L. maculans*-*L. biglobosa* species complex, the amount of non-repetitive DNA was very similar *ca*. 28–29 Mb (Table 1). This indicates that the size differences between genomes are essentially due to a difference in TE amount and not due to expansion or loss of gene families, as found in other fungi [33, 34]. Gene annotation predicted a comparable number (10624 to 11691) of protein-encoding genes in all genomes (Table 1). For each genome, the gene features were also very similar in terms of mean gene length, mean coding sequence length or proportion of genes with introns (Additional file 5: Table S2).

### Chromosomal organisation and synteny

**Figure 2:**
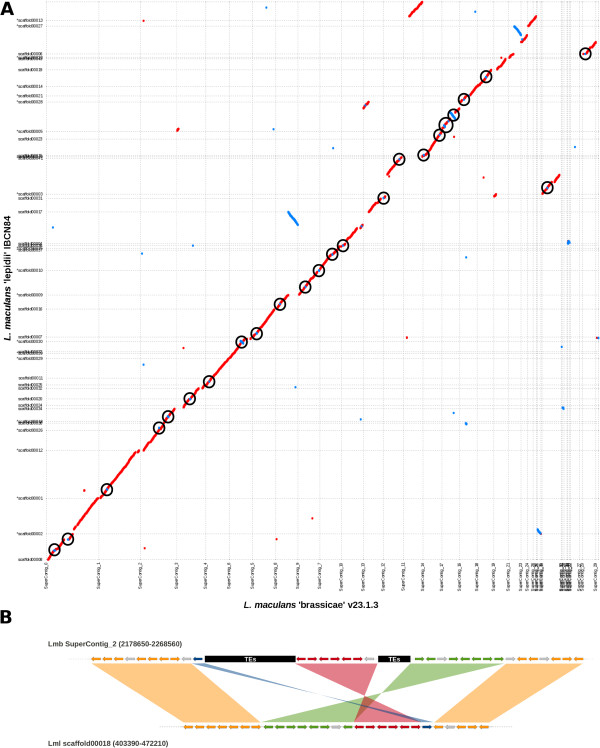
**Genome alignment and synteny analyses between** ***L. maculans*** **‘brassicae’** **(Lmb)** **and** ***L. maculans*** **‘lepidii’** **(Lml** **). (** **A)** Whole genome dotplot showing intrachromosomal inversions occurring in the genome of *L. maculans* ‘brassicae’ (horizontal axis) compared to that of *L. maculans* ‘lepidii’ (vertical axis). Inverted regions are circled in black and the number of inversions is mentioned close to the circle. **(B)** Schematic zoomed representation of one SuperContig_2 region (in Lmb) and its syntenic region in Lml. The region contains three inversions and the location of bordering transposable elements in Lmb are indicated as black boxes. The arrows represent genes and their orientation. Genes with no orthologs are colored in grey.

**Figure 3:**
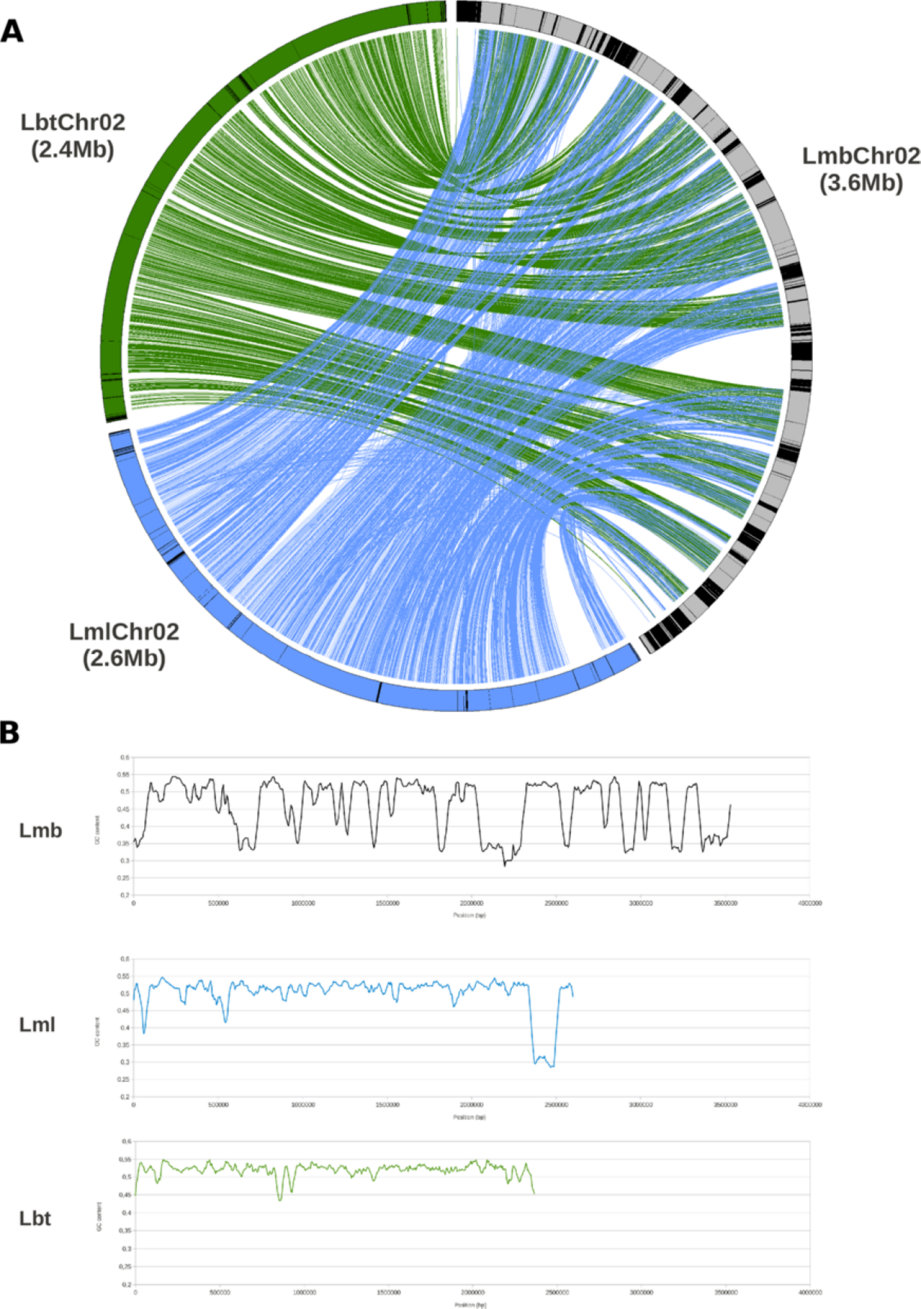
**Comparative chromosome structure in** ***L. maculans*** **‘brassicae’** **(Lmb),** ***L. maculans*** **‘lepidii’** **(Lml)** **and** ***L. biglobosa*** **‘thlaspii’** **(Lbt):** **the example of chromosome 2. (** **A)** Circos representation of synteny between the homologous chromosomes. The black parts represent the TE-rich, AT-rich chromosomal regions; **(B)** GC content changes along the chromosomes showing the typical isochore structure of the *L. maculans* ‘brassicae’ chromosomes, absent form the other species.

Lmb was estimated to have 17–18 chromosomes by combination of analysis of electrokaryotypes developed by PFGE (Additional file 1: Figure S1) and sequencing of the reference genome [9]. The new genomic data described here, especially those of the well-assembled genome of Lml (Table 1; Figure 2) and optical maps of Lmb, allowed this estimate to be refined by considering scaffolding errors on the reference assembly. With the support of genetic mapping, the chromosome count was updated to 19 (Additional file 6: Table S3), including the dispensable chromosome [15] only present in the two Lmb isolates. As a direct consequence to the overall GC-content of the genomes, the limited amount of TEs in Lml, Lbb, Lbc and Lbt resulted in a major difference in chromosomal structure: the AT-rich landscapes were mostly restricted to chromosome ends in Lml, Lbb or Lbt (Figure 3). Thus, within the species complex, only the Lmb genome had an isochore structure alternating large AT-rich and GC-equilibrated regions.

Repeat-masked nucleotide sequences of each genome were aligned with the others using MUMmer [35]. The genome alignment between the two Lmb isolates showed a perfect conservation of macrosynteny (Additional file 7: Figure S3A), as also observed by Ohm *et al*. [26] for isolates of *Cochliobolus heterostrophus*. Comparisons between Lmb and Lbb genomes, or between Lmb and Lbt genomes showed a globally well-conserved synteny with many intrachromosomal inversions (Additional file 7: Figures S3C, S3D). The alignment between Lmb and the most closely related species, Lml, is of particular interest since it showed a highly conserved macrosynteny pattern with only few major genomic rearrangements (Figure 2; Additional files 7 and 8: Figures S3B, S6). While large scale translocations were not seen, 30 intrachromosomal sequence inversions sized from 1.3 to 355 kb were identified (median size: 9.5 kb). These inversions were scattered along all the chromosomes and encompassed one to more than a hundred genes (Figure 2). The inversions within the chromosomes of the two isolates of Lmb analysed were bordered by TEs in 70% of the cases with TEs located at 82 bp on average from the inversion breakpoints (Additional file 9: Table S4; Figure 2).

The alignments also allowed to infer the sequence polymorphism between the different members of the species complex. The two Lmb isolates had very similar genomes, only 20,404 SNPs have been identified in the aligned non-repetitive regions (Table 2). In spite of highly syntenic genomes, the number of SNPs between Lmb and Lml was *ca*. 1.5 million. A similar number of SNPs between aligned regions is found when comparing other members of the species complex one to another, suggesting that what have been previously considered as sub-species might actually be distinct species.

**Table 2:** **Alignment length and SNP counts between the genomes of members of the** ***Leptosphaeria maculans***-***Leptosphaeria biglobosa*** **species complex**

		***L. maculans***‘brassicae’	***L. maculans***‘lepidii’	***L. biglobosa***‘brassicae’	***L. biglobosa***‘canadensis’	***L. biglobosa***‘thlaspii’
		Genome alignment length (Mb)				
***L. maculans*** **‘brassicae’**	No. of SNP	20404	24.2	8.1	8.4	8.6
***L. maculans*** **‘lepidii’**		1598215	-	8.2	8.6	8.7
***L. biglobosa*** **‘brassicae’**		1095420	1104219	-	25.9	15.6
***L. biglobosa*** **‘canadensis’**		1134785	1153336	1459160	-	16.3
***L. biglobosa*** **‘thlaspii’**		1147859	1163826	1739473	1794939	-

### Repertoire of transposable elements

Even though TEs were highly degenerated by RIP, the use of the REPET pipeline followed by extensive manual annotation allowed identification and classification of a large proportion of the repeats present in the genomes of Lmb, Lml, Lbb and Lbt (Additional files 10 and 11: Tables S5, S6; Additional file 12: Supplementary Data 1). One hundred and twenty-one consensus sequences have been identified of which 69 (57%) were classified as Class I or Class II TEs (Additional files 10 and 11: Tables S5, S6). Of these, 40 represented copy variants of TE families identified in several genomes and grouped into 16 larger families while the 29 remaining families were invariant between species (regardless of presence of RIP mutations).

TEs represented *ca*. 30% of the Lmb genome but only 4% and less in the other genomes (Table 1). Nevertheless, despite the major differences in genome coverage between Lmb and related species, a similar number of TE families (~30) were identified in each of the annotated genomes (Additional files 10 and 11: Tables S5, S6). Class I elements were the most abundant in all genomes (39.5 to 50.1% of the repetitive fraction in Lml, Lbb, Lbc and Lbt genomes) and up to 83% of the repetitive fraction in the genome of Lmb (Table 3). Uncertainties remain due to the high proportion of non-categorized repeats in the genomes of Lml, Lbb, Lbc and Lbt (19.2-48.8%) and higher proportion of unresolved nucleotides in the assemblies compared to the reference Lmb v23.1.3 (Table 1). Regardless of these uncertainties, our data support the fact that Lmb is an exception in terms of TEs and further indicate that the expansion of Class I TEs (mainly LTR retrotransposons) is remarkable in this species compared to related ones (Table 3).

**Table 3:** **Transposable element content in genomes of isolates of the** ***Leptosphaeria maculans***-***Leptosphaeria biglobosa*** **species complex**

		***L. maculans***‘brassicae’		***L. maculans***‘lepidii’		***L. biglobosa***‘brassicae’		***L. biglobosa***‘canadensis’		***L. biglobosa***‘thlaspii’	
TE class	TE order	Bases covered	% of repetitive fraction	Bases covered	% of repetitive fraction	Bases covered	% of repetitive fraction	Bases covered	% of repetitive fraction	Bases covered	% of repetitive fraction
Class I	Ty1-Copia LTR	3372500	22.1	77794	7.6	166805	14.9	34236	3.8	141432	10.0
	Ty3-Gypsy LTR	8749750	57.4	426573	41.7	368819	32.9	399244	44.4	365617	25.7
	LINE	6996	0.0	6523	0.6	15680	1.4	4535	0.5	54520	3.8
	Misc.	586134	3.8	1552	0.2	0	0.0	0	0.0	0	0.0
	*Sub*-*total*:	12715380	83.3	512442	50.1	551304	49.2	438015	48.7	561569	39.5
Class II	Tc1-Mariner	36075	0.2	193492	18.9	157676	14.1	76864	8.5	136579	9.6
	hAT	79154	0.5	1845	0.2	71796	6.4	21781	2.4	16950	1.2
	Mutator	890076	5.8	22524	2.2	42470	3.8	1344	0.1	3957	0.3
	MITE	0	0.0	11065	1.1	462	0.0	0	0.0	0	0.0
	Misc.	336819	2.2	19579	1.9	81576	7.3	30251	3.4	8034	0.6
	*Sub*-*total*:	1342124	8.8	248505	24.3	353980	31.6	130240	14.5	165520	11.6
Non-categorized	Non-categorized	1199008	7.9	262241	25.6	215260	19.2	331133	36.8	694332	48.8
	*Total*:	15256512		1023188		1120544		899388		1421421	

### Evolutionary dynamics of transposable elements

**Figure 4:**
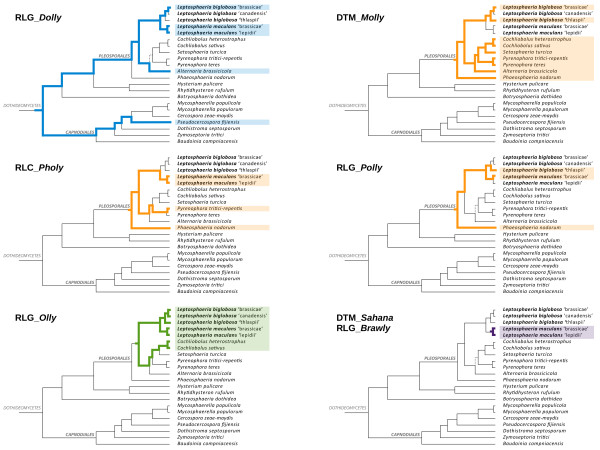
**Distribution of selected Transposable Elements** **(TE)** **within the Dothideomycete phylogeny.** The phylogeny is a simplified version of that in Figure 1. Rxx and Dxx correspond to retrotransposons and DNA transposons families respectively. The species in which the family has been identified are highlighted along with the corresponding branches. Colors indicate different times of invasion and are similar to those in Additional file 14: Figure S4.

Previously, using an alignment-based phylogenetic approach with TE sequences deprived of the RIP-affected sites, we postulated that TE expansion in Lmb genome took place between 4 and 20 MYA for TEs such as DTM_*Sahana*, RLG_*Rolly*, RLG_*Olly*, RLG_*Rolly* and RLC_*Pholy* while others such as RLG_*Dolly* had been resident and maintained in the genomes for very long times (>100 MYA) [9]. Mining for TE sequences in the entire *Ascomycota* phylum showed that TEs homologous to those present in the species complex were only found in class *Dothideomycetes* and were restricted to the order *Pleosporales*, with two exceptions, RLG_*Dolly* and DTT_*Krilin*, found in *P. fijiensis* a member of order *Capnodiales* (Figure 4; Additional file 13: Supplementary Data 2). As mentioned previously, this species, like Lmb, is TE-rich and has a low GC-content (Additional file 4: Table S1).

According to the chronogram in Figure 1, these two TE families have been present in the dothideomycete lineage for at least 300 MYA. This assumption is consistent with previous data that suggested RLG_*Dolly* as the most ancient TE present in the Lmb genome [9]. However it remains uncertain whether these two TE families invaded the fungal genomes before the *Capnodiales*-*Pleosporales* separation and then were widely lost in the *Capnodiales* or if their presence only in *P. fijiensis* is incongruent with vertical inheritance.

The remaining TE families are likely to represent surges of genome invasion at different times before and after the divergence of the *Pleosporales* (Additional file 14: Figure S4; Additional file 13: Supplementary Data 2). Among all the TE families identified in the *L. maculans*-*L. biglobosa* species complex, 66% are specific to the species complex (Additional file 14: Figure S4; Additional file 13: Supplementary Data 2).

Class I elements often showed an erratic phylogenetic distribution and presence/absence patterns in terminal nodes or terminal branches of the phylogeny. In contrast, Class II elements generally were better conserved amongst the *Pleosporales*. In addition, complete copies of DNA transposons were often maintained in all species in which they were present while only truncated copies or small-sized remnants were present for many Class I elements (Additional file 13: Supplementary Data 2). For example, DTT_*Molly* (previously identified in *Phaeosphaeria nodorum* [36]) was present in all species of *Pleosporales* except the *L. maculans* lineage (Figure 4). In many cases, two closely related species were missing one well-conserved TE family, further exemplifying the highly dynamic gain and loss patterns of TEs (*e.g*. DTT_*Finwe* absent from Lbt, but present in other members of the species complex, or DTA_*Kami* absent from *C. sativus*, but present in *C. heterostrophus* (Additional file 13: Supplementary Data 2)). Lastly, some TE families (*e.g*. DTT_*Yamcha* in Lml and *C. heterostrophus*, or DTF_*Elwe* in Lmb and *P. tritici*-*repentis*) were only found in distantly related species of the *Leptosphaeriaceae* and *Pleosporaceae*. This indicates multiple losses, independent invasion of the genomes or horizontal transfer events (Additional file 13: Supplementary Data 2).

### Transposable element dynamics in *L. maculans*‘brassicae’

Fifty one families, mainly corresponding to non-categorized repeats, were specific to terminal branches of the species complex. The number of these families varied from *ca*. 10 for the TE-poor genomes to 18 for the TE-rich Lmb genome, thus supporting the hypothesis of recent invasion of the Lmb genome by families of TEs absent from other members of the species complex. The presence of Lmb-specific TE families such as RLx_*Ayoly* (covering 400 kb of the genome of Lmb v23.1.3) and RLG_*Rolly* (covering 2.2 Mb) [9] (Figure 4; Additional files 1 and 8: Figures S1, S6) indicate a recent invasion of Lmb genome followed by a massive expansion of these families. Comparisons of the TE mosaics between the two isolates of Lmb showed that 61.5% of them are conserved indicating that this massive TE expansion occurred before, or at the onset of intraspecies divergence. In contrast, some families like DTM_*Sahana* showed highly diverse insertion patterns with only 12% of identical locations in the two Lmb genomes (Additional file 15: Table S7). This strongly suggests, as previously postulated [9] that DTM_*Sahana* is one of the most recent genome invaders and that waves of transposition activity took place after separation between the two Lmb isolates.

This simple picture indicating coincidence between genome invasion by new TE families and speciation becomes less straightforward when considering the three retrotransposons that account for most of the v23.1.3 genome expansion along with RLG_*Rolly*: RLC_*Pholy* (covering 3.1 Mb), RLG_*Polly* (covering 3 Mb) and RLG_*Olly* (covering 3 Mb) [9]. These three retrotransposons have been present in the *Pleosporales* phylogeny for at least 90 MYA, but they only represent a little fraction of the genomes of other *Pleosporales* species or other members of the species complex to-date (Figure 4; Additional file 13: Supplementary Data 2).

Classification of the TEs in families and phylogenetic analyses further indicated that the TEs associated with intrachromosomal inversions (Additional file 9: Table S4) between Lmb and Lml were mainly *L. maculans*- and even Lmb-specific TEs, *i.e*. recent TE-invaders such as DTM_*Sahana* or RLx_*Ayoly* (Additional file 9: Table S4).

### Gene conservation

**Figure 5:**
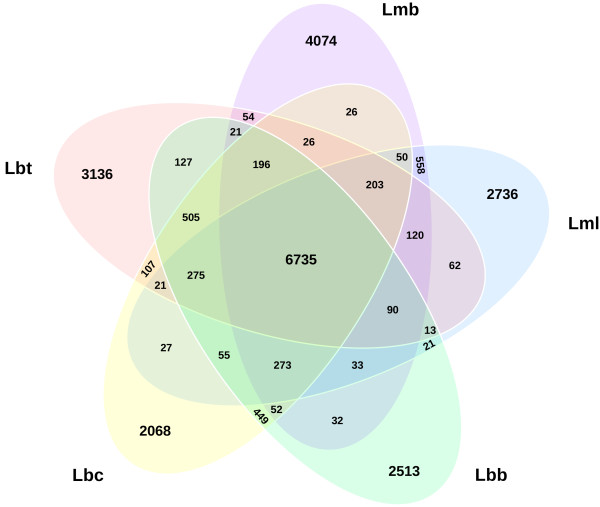
**Conservation of protein**-**encoding genes in the** ***Leptosphaeria maculans-*** ***L. biglobosa*** **species complex.** Lmb, *L. maculans* ‘brassicae’; Lml, *L. maculans* ‘lepidii’; Lbb, *L. biglobosa* ‘brassicae’; Lbt, *L. biglobosa* ‘thlaspii’; Lbc, *L. biglobosa* ‘canadensis’.

Of the 57,964 proteins predicted in the *Leptosphaeria* species complex, 75% were grouped into 10,131 families using orthoMCL [37]. Then, the core proteome of the sequenced species, encompassing 6735 proteins, was established (Figure 5).

To identify species-specific proteins, all the proteins that were not grouped into the 10,131 families were compared by BLAST at the nucleotide (BLASTn) and protein level (tBLASTn) against the other genomes and at the protein level (BLASTp) against the set of ungrouped proteins of the other species. The analysis allowed us to discriminate three classes of protein sequences: (i) species-specific sequences, (ii) sequences present in at least one other species and (iii) sequences for which presence or absence remained unresolved with our criteria (Additional file 16: Table S8). In addition, the occurrence of pseudogenes was investigated when lack of matching predicted protein sequence was associated with BLASTn or tBLASTn hits (Additional file 16: Table S8).

Each genome shared on average 10,400 proteins with (at least one of) the others, which represents 84.4-93.7% of the predicted proteins depending on the species. Usually, these sequences were conserved in organisms outside of the species complex since 74.3-85.5% of them have homologies in the NR database or harbour known protein domains. The remaining species-specific proteins, which represented 4.8-10.7% of the genomes (Additional file 16: Table S8) had very few predicted functions since 93.6-97.8% of them had no automated functional annotations. As a consequence, explaining species-specific biological or pathogenicity traits was not straightforward. However, two categories of species-specific genes could be identified and are analysed in more details below: genes encoding putative effectors and cluster of genes encoding secondary metabolites.

Interestingly, the Lmb genome was more enriched in specific sequences than all other members of the species complex and these genes were located in AT-isochores: 25.6% of the 620 genes of Lmb located in AT-isochores were species-specific, compared to only 9.8% of the genes located in GC-isochores.

### Pathogenicity gene analysis: effectors and secondary metabolites

#### Candidate effectors (small secreted protein)

The number of Small Secreted Proteins (SSPs) amongst the whole predicted proteome of each genome was similar in all the sequenced *Leptosphaeria* genomes, ranging from 621 in Lbc to 737 in Lml. They represented *ca*. 6% of the predicted proteins and 60% of the predicted secretome of each genome. Their features (153 amino acids on average; 2.8 times as rich in cysteine residues as other proteins in the genomes) were very similar in all *Leptosphaeria* isolates (Table 4). Their encoding genes were scattered along the chromosomes and had a similar GC-content (~52%) not different from the other predicted genes of the genomes (Table 4). However, as previously found, GC content varied as a function of genome location, and SSP genes in AT isochores had an average 48.2% GC content [9].

**Table 4:** **Characteristics of genes encoding Small Secreted Proteins** (**SSPs**) **and other predicted proteins in isolates of the** ***Leptosphaeria maculans***-***Leptosphaeria biglobosa*** **species complex**

	***L. maculans***‘brassicae’	***L. maculans***‘lepidii’	***L. biglobosa***‘brassicae’	***L. biglobosa***‘canadensis’	***L. biglobosa***‘thlaspii’
No. of SSPs	651	737	665	621	676
SSPs mean size (aa)	155.9	144.8	153.8	157.4	152.9
All predicted proteins mean size (aa)	416.5	427.4	434.6	464.2	421.0
%Cys in SSPs	3.10	2.75	2.60	2.86	2.79
%Cys in all predicted proteins	1.69	1.46	1.45	1.42	1.48
%GC in SSP genes	53	51	53	52	53
%GC in all predicted genes	53	53	54	53	54
RIP index^a^ in SSP genes	1.22	1.23	1.15	1.04	1.16
RIP index in all predicted genes	1.04	1.02	1.01	0.89	1.01
% TpA/ApT > =1.5 in SSP	18.4	19.4	13.5	8.2	15.5
% TpA/ApT > =1.5 in all predicted genes	7.0	5.0	4.1	1.7	4.2

^a^Repeat Induced Point mutation (RIP) index as measured by the TpA/ApT ratio.

The repertoire of SSP-encoding genes appeared to be in part extremely plastic and its conservation was consistent with phylogenetic distance with 73.8% of the SSP-encoding genes of v23.1.3 conserved in the other Lmb isolate, WA74, 54.2% conserved in Lml, and 42.5 to 44.0% conserved in *L. biglobosa* isolates (data not shown). In the core proteome, 20% of SSPs have a predicted function, and may relate to factors linked with pathogenicity such as carbohydrate-degrading enzymes (CAZys). However, the greater was the species-specificity, the less obvious was the potential function of SSP-encoding genes.

SSP-encoding genes did not generally occur as multigene families in the *L. maculans*-*L. biglobosa* complex, but a few of them (less than ten) had paralogs, usually conserved in all genomes. They corresponded to proteins usually belonging to multigenic families such as CAZys and were mainly located in GC-isochores in the Lmb genome. Only one effector gene with avirulence activity had an easily recognisable paralog in the Lmb genome: *AvrLm4*-*7* located within an AT-isochore had a paralog located 30 kb upstream on the same chromosome.

#### Avirulence effectors

Seven SSP genes conferring an avirulence phenotype on a series of genotypes of *Brassica* spp., *i.e*. interacting in gene-for-gene interactions with the plant surveillance machinery, have been cloned in Lmb. All are located in AT-isochores, and they generally showed a high level of diversification compared to their closest homologs (rarely above 30% sequence identity at the amino acid level), while usually maintaining a specific pattern of cysteine spacing (Additional file 17: Figure S7). They mostly showed a patchy distribution along the phylogenies or were specific to a few members of the species complex. Six of these had recognisable homologs in other fungal genomes:

*AvrLmMex* (A. Degrave, unpublished data) and *AvrLm11* [15] are specific to the *Leptosphaeriacae*, with *AvrLmMex* having homologs in every member of the species complex except Lml (Additional file 18: Table S9). The *AvrLm11*-encoding gene is located on a conditionally dispensable chromosome (CDC) present in some isolates of Lmb and absent from other isolates of the species complex [15]. AvrLm11, however, showed homologies with a SSP predicted in the Lbt genome (Additional file 18: Table S9) while three additional genes of the CDC also had orthologs in the genome of Lbt. These three genes were grouped in the Lbt genome at a different location to that of the *AvrLm11* homolog.

Sequences related to AvrLm1 [12] and AvrLm4-7 [14] were in a few species of class *Dothideomycetes* only. An AvrLm1 homolog was in the set of predicted proteins of Lbt but not in other members of the species complex (Additional file 18: Table S9). Interestingly, the orthologous gene was also located in a large but poorly assembled AT-rich region, devoid of other predicted genes. Both AvrLm1 and its Lbt ortholog showed homology with two SSPs with unknown function in the *Pleosporales* species *Pyrenophora teres f. teres* (Additional file 18: Table S9).

As described above, *AvrLm4*-*7* had a paralog in the Lmb genomes (*LmCDS2*, 65.5% of identity at the nucleotide level). This duplication did not occur in the other members of the species complex since either *AvrLm4-7* or *LmCDS2* had one-copy homologs in these genomes (Additional file 18: Table S9). Outside of the species complex, AvrLm4-7 or LmCDS2 had homology with two SSPs with no predicted function of the *Botryosphaeriales* species *Macrophomina phaseolina* (Additional file 18: Table S9).

In contrast to the previous examples, *AvrLm6* [13] and Lema_P086540.1 (A. Degrave, unpublished data) had homologs outside of the *Dothideomycetes*, but only in a few species of class *Sordariomycetes* (Additional file 18: Table S9). Within the species complex, these two proteins had SSP orthologs in Lml and Lbt (Additional file 18: Table S9).

#### Secondary metabolite gene clusters

**Figure 6:**
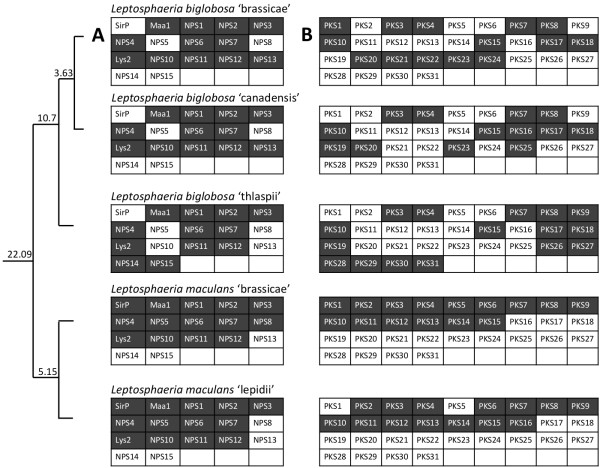
**Conservation of key secondary metabolite biosynthesis genes in the** ***Leptosphaeria maculans*** ***-L. biglobosa*** **evolutionary series.** **(A)** Non-ribosomal peptide synthases (NPS); **(B)** Polyketide synthase (PKS) genes. The colour of shading indicates those genes present (dark shading) or absent (white) in the corresponding species. The phylogenetic tree on the left is a simplified version of that in Figure 1, and the calculated divergence times (in MYA) are indicated.

Similar to genes encoding effectors, secondary metabolite biosynthetic genes such as Non-ribosomal Peptide Synthase (NPS) and Polyketide Synthase (PKS) genes showed examples of extreme specificity of occurrence and complete conservation within the species complex. NPSs and PKSs are multimodular genes that are keys in the biosynthesis of secondary metabolites. These genes are usually organised in clusters that include genes encoding enzymes such as methyl transferases, cytochrome P450 monooxygenases, hydroxylases that modify pathway intermediates. A total of 17 NPS genes were identified across the five members of the species complex (Figure 6A, Additional file 19: Table S10) and most (10) were shared by all five. *SirP*, the NPS involved in the production of the epipolythiodioxopiperazine toxin, sirodesmin PL [38], is only found in Lmb and Lml, which is relevant with biological data since *L. biglobosa* spp. do not produce this toxin. Globally, the distribution of NPS was well conserved between species in final branches (Lmb-Lml and Lbb-Lbc), whereas Lbt had an intermediate pattern of NPS. The species-specific NPS-genes in the complex may be the results of horizontal gene transfer or newborn pathogenicity determinants (Additional file 20: Supplementary Data 3).

Unlike the situation with NPS genes, there was a high degree of diversity in number and types of PKS genes identified across the members of the species complex (Figure 6B, Additional file 21: Table S11). In addition, and except for the examples mentioned below, homologs of PKSs were rare or absent in other *Dothideomycetes* and often had closest relatives in *Sordariomycetes* such as *Fusarium* spp. or *Colletotrichum* spp., or in *Aspergilli* (Additional file 20: Supplementary Data 3). A total of 31 PKS genes were identified of which only six were found in all species. Lmb and Lml had a very similar complement of PKS genes with 15 PKS in Lmb and only two missing in Lml, one of which was truncated. Twenty-three different PKSs were in *L. biglobosa* species and several of them were species-specific: six were only found in Lbt which reflects the distinctiveness of Lbt from other members of the species complex (Figure 6B).

The PKS and NPS biosynthetic gene clusters conserved between Lmb and Lml and between Lbb and Lbc displayed a high level of synteny (data not shown). Of the 16 clusters conserved in all the sequenced members of the species complex, eight were embedded in highly syntenic regions. This included genes widely conserved in the *Dothideomycetes* (*PKS4*, *PKS7*, *PKS10*, *NPS4*, *NPS6*, and *Lys2*), and also *NPS1* and *NPS11* that are only conserved within the species complex. In the eight other cases, secondary metabolite clusters showed microsynteny in Lmb and Lml, while they were reorganized in Lbb and Lbc. Once again, Lbt showed an intermediate genomic pattern with some clusters being syntenic with those present in Lmb and Lml (*e.g. PKS3* or *PKS15*) and some clusters showing microsynteny with Lbb and Lbc (*e.g. PKS8* or *PKS9*; data not shown).

**Figure 7:**
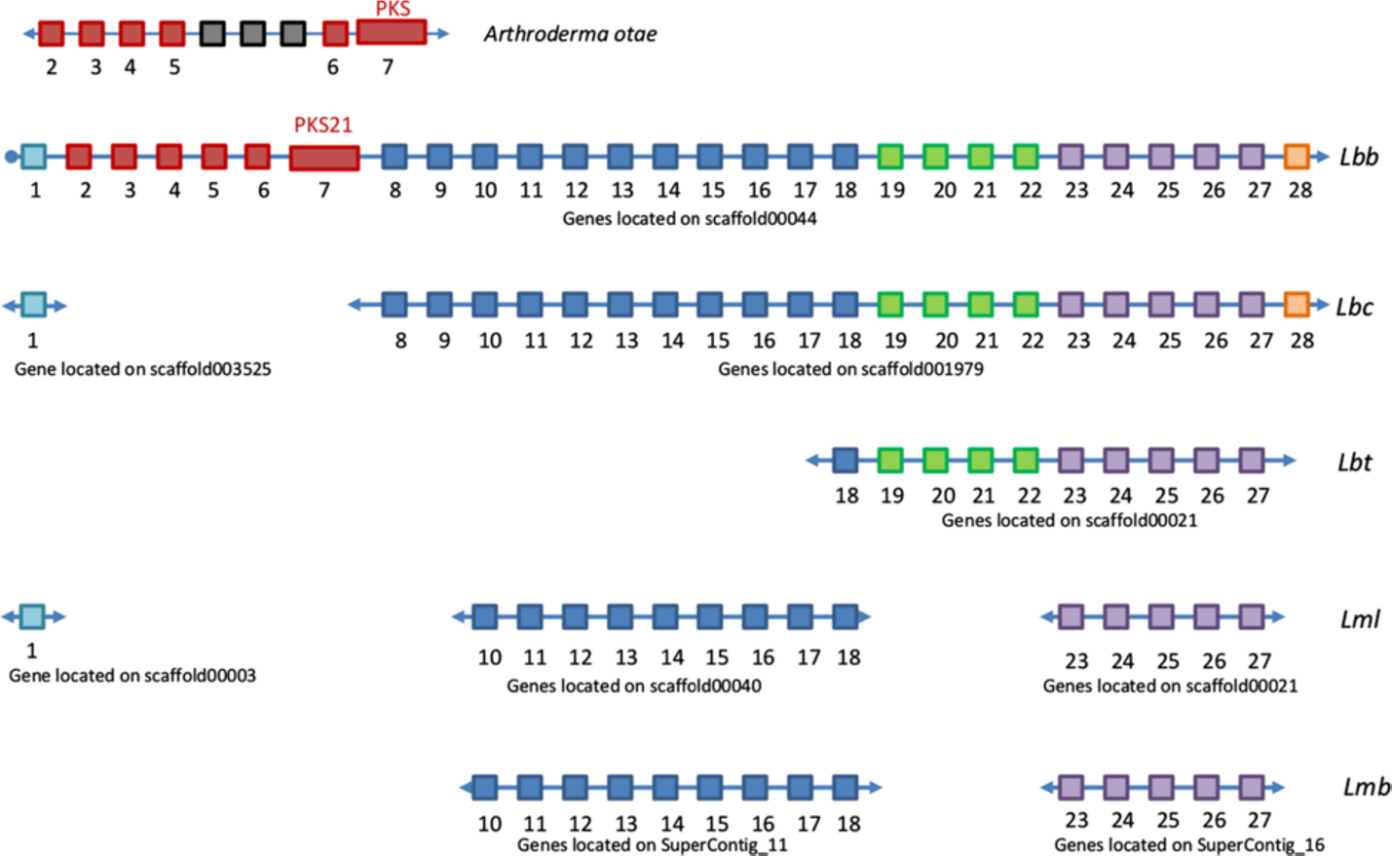
**Organisation of the genes surrounding the polyketide synthase,** **PKS21,** **of** ***L. biglobosa*** **‘brassicae’.** PKS21 and upstream genes show > 90% identity with genes from *Arthroderma otae*. Each square represents a single gene (not to scale). Genes that are present in different species are highlighted in the same colour. Ends of contigs are represented with a circle whilst arrows indicate that the contig continues. Lmb, *L. maculans* ‘brassicae’; Lml, *L. maculans* ‘lepidii’; Lbb, *L. biglobosa* ‘brassicae’; Lbt, *L. biglobosa* ‘thlaspii’; Lbc, *L. biglobosa* ‘canadensis’. Genes common to Lbb and *A.otae* have the following predicted functions: gene 2 has a retinol dehydrogenase (short chain dehydrogenase) motif; genes 3 and 5 have a methyltransferase motif, gene 4 has no conserved domains and gene 6 is a dimethyl allyl transferase.

Horizontal gene transfer has been proposed for the discontinuous distribution of several fungal secondary metabolite gene clusters [20, 39] and a clear example of this is seen in the situation of *PKS21*. Amongst the five species, this PKS was only present in Lbb, and reciprocal best matches showed that it had > 90% identity at both the nucleotide and protein level to a PKS from a very distantly related fungus, the eurotiomycete, *Arthroderma otae*, which is a dermatophyte (Figure 7). The five upstream genes showed high sequence similarity (>88% amino acid identity and 83% nucleotide identity) to genes upstream of the *A. otae* PKS, although this microsynteny was interrupted by the presence of three genes in *A. otae*. Several of the homologous genes had predicted functions consistent with biosynthesis of a secondary metabolite (Additional file 22: Table S12). Genes downstream of *PKS21* in Lbb were also present in genomes of some of the other *Leptosphaeria* species (Figure 7).

### Transposable elements and pathogenicity genes

Of the 620 genes located in AT-isochores in the Lmb genome, 148 genes (24%) were surrounded by TEs (within 2 kb of 5’ and 3’ ends of genes), whereas the others were located at the transitional regions between AT- and GC-isochores. Among these genes, 45 were conserved in other members of the species complex. In order to know if these genes were already present at the same location before the TE expansion, we investigated whether the region containing these genes and their closest flanking genes in Lmb were syntenic in the other genomes. For six genes, this analysis was impossible due to scaffolding differences of the assembly of the other genomes. For the remaining 39 genes, 26 did not encode SSPs and 81% of them showed a conserved microsynteny in the other genomes of the species complex, suggesting that the TE expansion did not influence the organisation of these small regions. Among the 13 genes encoding SSPs, six genes (46%) were not colocalized with their flanking genes while these latters were, suggesting that those six genes were translocated within the genome. Strikingly, five of these six translocated genes were characterized avirulence genes: *AvrLm6*, *AvrLm4*-*7*, *AvrLm11*, *AvrLmMex*, and Lema_P086540.1. Furthermore, a sixth avirulence gene, *AvrLm1*, was present in the 45 genes analyzed but could not be analyzed further due to poor assembly of its region in Lbt where it is likely to be hosted in an AT-rich genome environment.

Microarray and RNA-seq data were generated to identify the top 100 genes produced *in planta*, during the primary infection stage, by two species able to infect oilseed rape plants: Lmb and Lbb. Among the top 100 genes expressed at seven days post inoculation, 45% encoded SSPs in the Lmb genome, whereas this proportion decreases to 21% for Lbb (Additional file 23: Supplementary Data 4). Of these SSP-encoding genes, 30% represent species-specific genes in Lmb genome, with very few (or none) are species-specific genes in Lbb.

**Figure 8:**
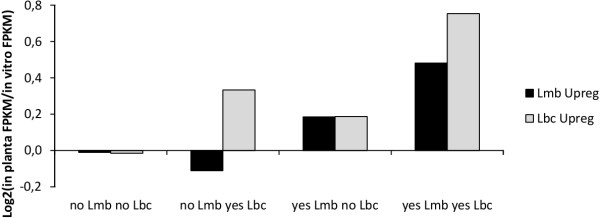
**Effect of presence of repetitive element adjacents to promoter of orthologs on gene expression** ***in planta*** **compared to in axenic culture.** *In planta* upregulation ratio (Log2(*in planta* FPKM/*in vitro* FPKM)) calculated from RNA-seq data for orthologs in *L. maculans* ‘brassicae’ (Lmb) and *L. biglobosa* ‘canadensis’ (Lbc) during growth *in planta* and *in vitro*. Ortholog pairs were categorised according to the presence or absence of repeat elements adjacent to the promoter region of both genes. Positive up-regulation ratios indicate higher expression during growth *in planta*. The categories were: no Lmb no Lbc (n = 6959), no Lmb yes Lbc (n = 60), yes Lmb no Lbc (n = 382), yes Lmb yes Lbc (n = 14).

RNA-seq gene expression values for Lmb and Lbc orthologs were coupled with proximity of TEs in the gene environment. Orthologous genes of Lmb and Lbc are more highly up-regulated *in planta* (on average) when a TE is located close to their promoter, than when it is not nearby (Figure 8).

## Discussion

In this study, we used comparative genomic approaches to reassess speciation issues in the *L. maculans*-*L. biglobosa* species complex and to evaluate the role of TEs in genome reshaping and the rise of species with improved pathogenic abilities. Our data demonstrate that (i) each entity of the species complex actually is a distinct species; (ii) TEs likely contributed to speciation by generating chromosomal inversions; (iii) TE invasions/burst are recent at the evolution scale; (iv) TEs contributed to build two-speed genomes and most likely to translocate effector genes within plastic regions of the genome, with consequences on gene diversification, better adaptation to selection pressure and gene expression. All in all, TE bursts and their consequence on genome structure favored adaptation and pathogenicity towards oilseed rape by allowing generation of novel effector genes and their concerted expression during the first steps of plant infection.

### Reassessing speciation within the *L. maculans*-*L. biglobosa*species complex

Fungi are estimated to include over 1.5 million species [40]. Their description and classification have been long based on macro- or microscopic criteria, such as morphology or reproduction mode. The availability of genomic data for a large number of fungal species has allowed the inference of new phylogenetic relationships between close or even unrelated taxa previously aggregated in the same genera. This is exemplified by the *Mycosphaerella* genera and its new classification [41, 42]. Here, we firstly re-investigated speciation issues in the *L. maculans*-*L. biglobosa* species complex based on sequence data analyses. The estimated divergence times between the terminal clades (5.1 MYA between Lmb and Lml and 3.6 MYA between Lbb and Lbc) are consistent with divergence time between species in closely related genera (*Cochliobolus sativus* and *C. heterostrophus*: 4.3 MYA, *Mycosphaerella populicola* and *M. populorum*: 4.7 MYA, *Coccidioides posadasii* and *C. immitis* 5.1 MYA [43]) (Additional file 2: Figure S2) and strongly indicate all *Leptosphaeria* isolates analysed here belong to different species. This hypothesis is supported by high numbers of SNPs (*ca*. 1.5 million) detected between each lineage of the species complex, which are in a comparable order of magnitude than those observed between different species of *Cochliobolus* [44] Further support for this hypothesis is provided by the degree of mesosynteny, *i.e*. the conservation within chromosomes of gene content but not order or orientation, between the members of the species complex [45]. Mesosynteny, firstly identified in *Dothideomycetes*, was recently postulated to be a mode of chromosomal evolution specific to fungi [45]. Comparative analyses of 18 dothideomycete species, and a simulation-based approach indicated that serial random inversions within the chromosome lead with time to extensive reshuffling of gene order within homologous chromosomes from one species to a distantly related one [26]. Focusing on relatively short divergence times, our analyses are consistent with this modelling-based hypothesis. The number of genome rearrangements (30) between Lmb and Lml is many fewer than what has been reported between *Cochliobolus sativus* and *C. heterostrophus*, where there were as many as 30 inversions in a single scaffold [26]. This represents an earlier step towards mesosynteny than what was described for the *Cochliobolus* species.

### TE regulation in ascomycete genomes

Sequencing confirmed that the genome of Lmb contains a larger proportion of TEs than those of the other members of the species complex, but also those of other *Pleosporales*. Heavily TE-invaded genomes can be found in ascomycetes such as *Blumeria* spp., in basidiomycetes or in oomycetes [8, 46]. Often related species all show heavily TE-invaded genomes as exemplified by downy mildew fungi or rust fungi [8, 46]. An intermediate situation is found in fungi in order *Capnodiales* of the *Dothideomycetes* that have a series of distantly related species with either TE-poor or TE-invaded genomes [26, 31]. Compared to these examples, *Pleosporales* are an exception since all species sequenced to-date, including those of the species complex sequenced here show TE-poor genomes and TE expansion is only observed in Lmb.

In *Phytophthora* spp. and downy mildew fungi, the absence of irreversible defense systems able to regulate the TE activity results in an evolutionary trade-off in which the fitness cost linked with genome “obesity” eventually counter-selects individuals for which the fitness deficit is higher than the selective advantage conferred by enhanced genome plasticity. Compared to the *Blumeria* or *Phytophthora* examples, the expansion of TEs in the genome of Lmb (as in that of many other ascomycetes) remains limited (36% of the genome for Lmb *vs*. 64% of the genome for *B. graminis* and 74% for *P. infestans* [8]). These data question about how TEs were controlled through time by Lmb and, in general, by most ascomycete species.

RIP is likely the most efficient genome defense mechanism against TEs and has been experimentally demonstrated to be currently active in *L. maculans* [47]. Evidences for past or current RIP activity has been reported in most ascomycete species, suggesting that RIP is an ancestral mechanism common to (at least) ascomycete fungi [48], with occasional patterns of secondary losses like observed in *Blumeria* spp. [33]. Since RIP induces irreversible changes in TEs, thus disabling their transposition mechanisms, it is therefore confusing how massive transposition activity could have taken place recently in Lmb genome for LTR retrotransposon families potentially present in the *Pleosporales* lineage since 100 millions years. Our data suggest that these TE families were not inactivated by RIP or that they were reactivated. Three main and complementary hypotheses, discussed below, could explain these data: (i) loss and regain of RIP activity with involvement of another epigenetic mechanism to control TE transposition events, (ii) biological traits preventing RIP activity, *i.e*. absence of sexual reproduction and (iii) recent and massive events of horizontal transfer (HT).

(i) Other inactivation mechanisms, also linked to the sexual cycle, have been described in Fungi such as methylation induced premeiotically (MIP) in the ascomycete *Ascobolus immersus* [49] and in the basidiomycete *Coprinus cinereus* [50]. MIP is an epigenetic mechanism which methylates but does not irreversibly mutate duplicated sequences. The fact that it is conserved between ascomycetes and basidiomycetes, raises the possibility that RIP evolved from MIP or from a similar silencing process [11]. The breakdown of such genome defense mechanism seemed to be linked with bursts of transposition in many species, resulting in genome rearrangements and eventually speciation [51]. Thus, we can hypothesize that a reversible epigenetic mechanism was previously active in Lmb genome and relieved under a stress-inducing event at the time of speciation, leading to the unleashing of transposition activity. The fact that all TE families are affected by RIP (none active TE copies in Lmb genome) suggests that epigenetic mechanisms were replaced by RIP as an irreversible control mechanism.

(ii) RIP is only active during meiosis, therefore prolonged growth in absence of sex would prevent RIP inactivation of TEs. Lmb undergoes prolific sexual reproduction [12], but asexual populations exist that are either adapted to other crucifers such as cabbage or present in environments where short growth season of the host plant prevent the fungus from completing its sexual cycle [52, 53]. Asexual growth could also be imposed within a founder population with only one mating type. We can thus hypothesize that the rise of the Lmb species was followed by a long phase of asexual behaviour in order to propagate rapidly the newly-born species, without preventing expansion of TEs until sexual reproduction became prevalent with the need to diversify genotypes to adapt changing environmental conditions. Under this hypothesis, the other members of the species complex known to also use profusely sexual reproduction (*e.g*. Lbb in Europe or Lbc in Australia) never had such a long phase of asexual behaviour and efficiently limited TE expansion using RIP.

(iii) TEs can invade a genome through HT [54, 55] and, in animals, arthropods vectors are allowing HT between species [55]. Microbes sharing the same diverse ecological niche with Lmb (soil, stem residues, leaf surfaces, plant tissues), but whose biodiversity is currently unexplored, or the host plants themselves are candidates vectors for such HT of TEs to Lmb genome. Our phylogenetic analyses did not allow us to establish whether such HT events occurred in the dothideomycete phylogeny, but multiple cases of patchy distribution of TE families do not contradict this hypothesis. In addition, even with RIP being active and occurrence of sexual reproduction, constant donation of TEs could occur from a donor that was present over an extended period of many million years, such as a virus or an intracellular symbiotic bacteria [56]. The donor TEs would not be affected by RIP, as it is protected in the vector, but each new introduction would be RIPed and inactivated as it arrived. Long term addition could result in the establishment and the expansion of TE-rich regions. We acknowledge that this is extremely speculative, as currently massive and recent introduction and expansion of TEs cannot be distinguished from multiple introductions, due to extensive sequence degeneracy following RIP.

### Speciation and TE expansion

In many eukaryotic lineages, links between bursts of transposition and speciation are observed, but it is difficult to determine whether these bursts were responsible for speciation or if they were a consequence of it [51]. In this study, two events were predicted to have taken place during the same period of time: (i) the speciation event separating Lmb from Lml and (ii) the introduction in the Lmb genome of new TE families followed by the expansion of ancient and recent TE famillies. The effects of invasion by species-specific TEs on speciation events are difficult to sort out since introduction of new TE families were observed several times during evolution within the dothideomycete lineage while bursts of transposition did not occur in any other *Pleosporales* species. However, the analysis of TE distribution was based only on TEs identified in the members of the *L. maculans*-*L. biglobosa* species complex, which precluded inference of relationships between TEs and speciation in the other *Pleosporales* genera. Moreover, as mentioned previously, the patchy distribution of some TE families, especially LTR retrotransposons, could be evidence of HT of TEs between species [57] and thus disrupt the evolutionary history of these elements. In Lmb 70% of the intrachromosomal inversions were bordered by TEs specific to the *L. maculans* clade. Ohm *et al*. [26] suggested that inversion breakpoints in dothideomycetes genomes were associated with Simple Sequence Repeats (SSR), a feature that was not apparent when comparing Lmb and Lml genomes. These data may indicate a major role for TEs rather than SSRs in genome reshaping and reshuffling at the chromosomal level, similar to the documented incidence of TEs on chromosomal inversions in more complex Eukaryotes such as *Drosophila* [58] or mammals [59]. This first step towards mesosynteny clearly ascribes a role to species-specific TEs in this evolutionary mechanism that will eventually generate non-homologous sections of chromosomes and isolate part of the genome from meiotic recombination.

### Can genome data explain biological and phytopathological specificities?

TEs are involved in adaptation of phytopathogens to new hosts by promoting HGT, as exemplified by the transfer of a host selective toxin, ToxA between *Phaeosphaeria nodorum* and *Pyrenophora tritici*-*repentis*, thus generating a new disease because of better adaptation of strains of *P. tritici*-*repentis* to their host plant, wheat [60]. TEs also promote the birth of new pathogenicity traits via gene duplication and translocation eventually generating large multigene families of effectors as exemplified by the downy mildew fungi and *Phytophthora* species [8, 33].

Our data were indicative of HGT between phylogenetically divergent species mostly for secondary metabolite gene clusters, of which some species-specific are located within AT isochores in Lmb, but information on the involvement of TEs in these HGT phenomena were sparse. Such HGT is seen in another member of the species complex, Lbb, in the situation of PKS21 and surrounding genes of the cluster only found in the eurotiomycete *A. otae*. The consequences of this on Lbb pathogenicity (if any) are not known. One other intriguing example is that of NPS8, the five-module NPS thought to be involved in the production of the transiently produced depsipeptide, phomalide. Phomalide is postulated to be an important pathogenicity determinant as it has been proposed to act as an host-selective toxin (HST) [61]. The gene cluster is located in an AT-rich subtelomeric region of the genome of Lmb and has no known homologs.

For SSP-encoding genes, indication of HGT were sparse due to high level of sequence divergence with possible homologs within or outside the species complex. We confirmed here that effector genes did not belong to recognisable multigene families, and that TEs are unlikely to promote multiple duplications. This is consistent with the known impact of RIP on restriction of gene innovation mediated by duplications [11]. However, in cases where homologs could be identified, another impact of TEs on SSP genes could be evidenced here for an important part of the effector genes known to be also behaving as avirulence determinants during plant pathogen interaction. Microsynteny analyses between the members of the species complex highlighted the fact that, in the Lmb genome, 5 of 7 characterized avirulence genes present in AT-rich isochores have been translocated from another location in the genome to these TE-rich environments in the course of evolution. This is reminiscent at a different scale of what is observed in *M. oryzae*, where a paralog of *Avr*-*Pita* (*Avr*-*Pita3*) is found at an invariant genome location, while *Avr*-*Pita* and other variants may be absent or present at different chromosomal locations, or on different chromosomes, depending on the isolate [62]. In *M. oryzae*, both patterns of complete loss of the gene and patterns of multiple translocations were ascribed to the close association of *Avr*-*Pita* with a retrotransposon. The authors postulated that other avirulence genes of *M. oryzae* may have the same behaviour and also be subjected to multiple translocations in the genomes and that this maintains a pool of isolates with the avirulence gene when selection is exerted so that it can be easily disseminated in populations when the selection is no more present. In our study, these translocations were not identified between Lmb isolates but between members of a species complex displaying contrasted TE-content in their genome. This suggests that during the TE expansion, genes (known today as effector genes) were subjected to TE-mediated translocations, resulting in many cases in their isolation within highly dynamic TE-rich compartments. We thus highlight here another mechanism from what is reported in species for which TE are drivers of gene innovation via generation of multigene families: in Lmb, probably because of the activity of RIP, duplication only rarely occurs, but TEs contribute to displace the genes of importance in novel dynamic regions of the genome.

On this basis the question is to understand what can be the selective advantage of displacing effector genes in such plastic genomic regions and how this contributes to enhanced pathogenicity towards oilseed rape. While being morphologically very similar [23, 63] and adapted to crucifer hosts, the more divergent species of the species complex, Lmb, Lbb and Lbc have very similar host range and pathogenic behaviour. They are pathogenic towards Brassica species and share most of their infection strategies and specificity of life cycle [64, 65].

Also, the divergence time which predate speciation (*ca*. 22 MYA) between *L. biglobosa* and *L. maculans* correlates well with current estimates of the whole-genome triplication events that facilitated the origin of *Brassica* crops centered at 22.5 MYA [66]. This could indicate the *L. maculans*-*L. biglobosa* lineage co-evolved with its host to become adapted to these newly-born triplicated *Brassica* plants and that adaptation to *Brassica* predates enhanced pathogenicity towards oilseed rape observed in Lmb. Under this hypothesis, the very restricted host range of Lml (nonpathogenic to *Brassica* species but specialised on *Lepidium* sp. [21, 63]) would be a secondary adaptation to a crucifer weed, possibly linked with its expanded repertoire of SSP-encoding genes compared to the other members of the species complex. In *M. oryzae*, effector repertoire is associated with host range expansion; loss of a single effector AVR-Co39 enables this fungus to colonize rice, as well as foxtail millet [67, 68]. Perhaps the expanded repertoire of effectors in Lml contributes to its narrower host range and its specialisation to *Lepidium* sp. compared to other members of the species complex.

While pathogenic strategies and life cycle characteristics have been conserved between Lmb and Lbb/Lbc for 22 million years, Lmb shows some pathogenicity traits that may have been acquired more recently. Two main differences exist between the species: aspects of leaf lesions and ability to develop the stem canker. According to the leaf symptoms they cause in the field or in controlled environments, Lbb and Lbc often have been considered as “weakly pathogenic” [21, 69]. However Lbb and Lbc often cause large lesions on cotyledons: Lmb causes grey tissue collapse with no visual evidence of plant responses, suggesting the fungus has deployed mechanisms to suppress plant defenses, while the *L. biglobosa* isolates cause limited tissue collapse containing numerous large-size black spots suggesting the presence of some plant defense responses [70, 71] (Additional file 24: Figure S8). Lmb is the most damaging pathogen of *B. napus* as it causes cankers at the base of the stem that cause the plant to lodge, resulting in significant losses at harvesting, while Lbc and Lbb cause limited upper stem lesions, of much lesser incidence on yield. These differences in pathogenic abilities were difficult to relate to gene content since most of genes, which are species-specific and thus potentially involved in species-specific life traits mostly did not have any attributed function. In contrast, genes with possible function in disease (*e.g*. CAZys and proteases that are responsible for fungal nutrition) were conserved between the different members of the species complex. In addition, the amount of SSP-encoding genes was comparable between the three species. However, most of the species-specific effector genes of Lmb expressed early during the plant infection are closely associated with TEs in the Lmb genome and located in large AT-rich isochores. It is possible that the displacement in AT-isochores resulted in accelerated sequence diversification of effector genes due to action of RIP on non-duplicated sequences hosted in the AT-rich environment [17, 18]. The lower AT content of these genes compared to that of other genes encoding SSPs in the GC isochores of Lmb [9] or of genes encoding SSP in other members of the species complex (this study) would be in favor of such an environment-mediated accelerated rate of diversification of the genes and thus explain the enrichment in species-specific effector genes in AT-isochores of Lmb.

Our data also indicate a link between proximity of TEs and *in planta* gene expression. Eventually, moving effector genes in the AT isochores would favor *in planta* expression of effector genes and since *ca*. 120 candidate effector genes are located in (or at the borders) of AT-isochores results in concerted expression of these at the onset of plant infection [9]. We previously showed that AT-isochores create heterochromatic landscapes responsible for an epigenetic control of the effector gene expression [72]. Here, we showed that patterns of expression of effector genes differ between Lmb and Lbb. Amongst the top 100 genes expressed following cotyledon at seven dpi, 45% encode SSPs in the Lmb genome, whereas this proportion decreases to 21% for Lbb (Additional file 23: Supplementary Data 4). The finding that 30% of the top 100 Lmb genes up regulated *in planta* represent species-specific genes located in AT-isochores, compared to very few in Lbb, suggests that the concerted expression of these species-specific SSPs in Lmb play a major role at the onset of the plant colonisation and may contribute to the development of the typical grayish-green tissue collapse on leaves, possibly by suppressing the plant PTI (Pathogen Triggered Immunity), a function commonly attributed to effector genes expressed in the earliest stages of plant infection [73]. The Lmb-Brassica co-evolution and the early expression of effector genes *in planta* to suppress PTI eventually allowed the plant to build a second line of defense response, Effector Triggered Immunity (ETI) that seems to occur in the Lmb-*B. napus* interaction, but not in the interaction with Lbb or Lbc [64]. As a consequence, gene-for-gene interactions are observed in the Lmb-Brassica interactions, but not in interactions of Brassica species with *L. biglobosa* species. In contrast to the Lmb case, the dark lesions caused by Lbb or Lbc ([71]; Additional file 24: Figure S8) suggest that the plant initiates PTI and that the fungus cannot efficiently suppress it. In this case, speed of growth of the fungus and multiple re-infection points nevertheless allow more or less efficient subsequent tissue colonisation.

Apart from an extensively described role of AT-isochores in rapid adaptation to plant resistance selection [16, 18, 19], and probable role of HGT, TEs thus are likely to have played a major role in the course of evolution by moving effector genes to novel plastic genomes environments promoting sequence diversification and concerted expression of effector genes at the onset of plant infection, eventually resulting in the success of Lmb as a destructive pathogen of oilseed rape and other Brassicas.

## Conclusion

In fungi, genome invasion by TEs is often postulated to have contributed to the success of phytopathogens, mutualists and endophytes and, as stated by Raffaele & Kamoun [8], phytopathogens often have expanded genomes compared to their free-living relatives. The *L. maculans*-*L. biglobosa* system also shows that TEs have contributed to intrachromosomal inversions, likely resulting in speciation as already described for more complex Eukaryotes. In Lmb, as in other phytopathogens, the hosting of effector genes in TE-rich genome environment contributed to sequence diversification and probably adaptation to new hosts or better adaptation to the existing host. It currently contributes to a highly dynamic plasticity and diversification of these genes. In Lmb, RIP mutation increases the speed with which the detrimental genes can be mutated to extinction, rendering migration of low importance compared to the ability to generate variants locally in a single sexual cycle. To-date, this process has not been documented in other fungal species. The hosting of effector genes in such dynamic genome environments has a dual advantage for phytopathogens: immediate adaptation to a new source of resistance (provided the effector gene is dispensable) consistent with the speed of birth and dissemination of new virulent populations, and long-term enhanced ability to duplicate and diversify effector genes to facilitate adaptation to the host or adaptation to new hosts. Recent advances suggested that the association of effector genes with TEs also has an impact on epigenetic, heterochromatin-based regulation of their expression. This, along with evolutionary consequences of this finding, provide a new and promising field for research.

## Methods

### Biological material

Isolates IBCN84 (*L. maculans* ‘lepidii’) and IBCN65 (*L. biglobosa* ‘thlaspii’) are part of the International Blackleg of Crucifer Network collection maintained at INRA-Grignon and AAFC Saskatoon, Canada. Both isolates were obtained as hyphal tip-purified isolates in Saskatchewan either from stinkweed (*Thlaspi arvense*) (IBCN65) or from peppergrass (*Lepidium sp*.) (IBCN84) [21]. Isolate B3.5 (*L. biglobosa* ‘brassicae’) is one of two isolates that were used to formally establish the new *L. biglobosa* species name [30]. It was obtained as a single-ascospore isolate ejected from a pseudothecia formed on *Brassica juncea* cv. Picra in an experimental field at INRA-Le Rheu [30]. The *L. biglobosa* ‘canadensis’ isolate 06VTJ154 (hereafter named J154) was cultured from ascospores released from sexual fruiting bodies on *B. juncea* stubble collected from Burren Junction, New South Wales, Australia as described by Van de Wouw *et al*. [70]. The *L. maculans* ‘brassicae’ isolate WA74 was purified from infected stubble of oilseed rape collected from Western Australia. It is also known as IBCN76 [21]. The Lmb isolate used for RNA-seq experiments was IBCN18, an Australian isolate cultured from infected stubble of oilseed rape.

### Sequencing and assembly

For isolates IBCN84 (*L. maculans* ‘lepidii’), IBCN65 (*L. biglobosa* ‘thlaspii’) and B3.5 (*L. biglobosa* ‘brassicae’), a Paired-End 8 Kb library was constructed according to the protocol 454. A run and a half was done on the Titanium version, generating 580, 511 and 614 Mb of raw data, respectively. An additional Paired-End 20-kb library was built for isolate B3.5. An additional run was done on the Titanium version, generating 380 Mb of raw data. Additional libraries were constructed according to the Illumina protocol with an average size of 250 bp inserts for IBCN84 and IBCN65 and 350 bp for B3.5. Libraries were sequenced on three lanes (for IBCN84 and B3.5) and 2 lanes for IBCN65 on a GAIIx, 76 bp in single sequencing, generating 7.8 Gb of raw data for IBCN84, 6.08 Gb for B3.5 and 4.9 Gb for IBCN65. The Titanium sequences were assembled by Newbler and the sequences of the scaffolds were corrected using the Illumina sequences.

For isolate WA74, DNA was extracted (CTAB extraction protocol) from mycelia grown on V8 agar plates. Sequencing was done using Roche 454 Titanium platform both for shotgun sequencing and sequencing of an 8 kb mate-paired library. A total of 1,308,735 shotgun reads was obtained including 459,965 reads from the paired library prep where 248,433 were discernable pairs and had an average insert size of 7.5 kb. A *de novo* assembly of isolate WA74 was performed for the shotgun and paired library data using gsAssembler version 2.3 (Roche/454).

*L. biglobosa* ‘canadensis’ isolate J154 was grown in 10% Campbell’s V8 juice for five days and genomic DNA was extracted from mycelia and sequenced by the Australian Genome Research Facility, Melbourne, Australia using Illumina HiSeq2000. Libraries were constructed with a 250 bp insert size. A single lane of 100 bp pair-end sequencing was used to generate 32 Gb of raw data. Reads were trimmed to a minimum quality of phred 28 using FastX-toolkit software, producing 310 million trimmed reads. Trimmed reads were assembled de novo using the Velvet short read assembler v1.1.06 [74] with the Velvet Optimiser Perl script to select the k-mer (49), expected coverage (402) and coverage cutoff (43) values. The final assembly of *L. biglobosa* ‘canadensis’ J154 produced 29.48 Mb of scaffolds > 200 bp.

### Optical mapping of the v23.1.3 genome

The optical map of *Leptospheria maculans* ‘brassicae’ v23.1.3 was generated using the Argus Whole-Genome Mapping System (http://www.opgen.com). Restriction enzyme, *Mlu*I, was chosen using Enzyme Chooser (OpGen Inc., Gaithersburg, MD), which identifies enzymes that result in a 6–12 kb average fragment size and no single restriction fragment larger than 80 kb across the genome. Overall, 8915 single-molecule restriction maps with an average size of 248 kb were generated. The total size was ~2 Gb and 75 supercontigs were obtained after assembly. Fasta files were imported to the MapSolver software and converted into *in silico* maps by analysis of *Mlu*I restriction fragments. The MapSolver software then compared and reconciliated the different restriction maps.

### Bioinformatics

#### Gene annotation

The automated gene annotation was carried out using a combination of two *ab initio* gene predictors, Fgenesh [75] and Genemark [76]. Fgenesh has been previously used as a part of the EuGene pipeline [77] for annotation of the *L. maculans* ‘brassicae’ v23.1.3 genome [9]. Genemark was trained on the twenty largest SuperContigs of *L. maculans* ‘brassicae’ v23.1.3 with the repeated sequences masked using RepeatMasker [78] in order to avoid the RIP bias in base usage on the repeated elements. Both Fgenesh and Genemark were firstly benchmarked on one SC of v23.1.3 and showed that Genemark gene models were better defined than Fgenesh and EuGene gene models. As a consequence, Genemark predictions were always prioritized over Fgenesh prediction in case of inconsistent annotation between the two predictors. Both Fgenesh and Genemark were run on repeat-masked genomic sequences and the results were combined. Gene models encoding proteins > 30 amino acids were compared and a decision made as follows: (i) if a similar gene model was predicted at the same locus, or if it was predicted by one or the other of the predictors, this gene model was kept; (ii) if two different gene models were predicted at the same locus, the Genemark prediction was prioritized; (iii) if a predicted gene model corresponded to two or more gene models from the other predictor, the latter was kept. All validated genes were then translated into proteins and their features were written in a GFF3 formatted file which was used for visualisation (*e.g*. with Artemis [79]).

#### Functional annotation

Automated functional annotation of the predicted proteins was performed by using a combination of BLAST [80] and InterProScan [81]. First, each protein was compared by BLASTp to the NR database, and only the hit results matching the following criteria were kept for further analysis: (i) the e-value should be less than 1e-06, (ii) the similarity percentage should be over 30%, (iii) at least 70% of the query sequence should be covered by the alignment length, (iv) the subject (hit) sequence length should represent between 75 and 125% of the query sequence length. Then, all validated results were pooled and average values were calculated for each of the above-mentioned criteria. A consensus description was then obtained by text-mining. According to these different features, proteins were divided into three classes: (i) proteins with no BLAST hit result or BLAST hit results with a mean percentage of similarity lesser than 40%, a mean percentage of coverage of the query sequence length by the alignment length lesser than 80% and a mean percentage of coverage of the query sequence length by the subjects length lesser than 85% or greater than 115%, and (ii) proteins with no protein domain identified by InterProScan. This class is classified as « predicted protein », i.e., predictions with no functional support. It usually contained species-specific sequence with no known domains. The second class, termed « hypothetical proteins », included predictions that (i) had at least one domain identified by InterProScan in the InterPro database [82] and fulfill the above-mentioned BLAST result criteria but for which text mining indicated « hypothetical protein » in more than 90% of the hits, or (ii) fulfill the BLAST results conditions with a consensus description corresponding to a function but with no protein domain identified, or (iii) with no BLAST results but with at least one domain identified from the InterPro database. Globally, this class contained predictions with slight functional support that might correspond to conserved proteins among several organisms but with no defined functions, or to splitted/merged predictions. The third class included predictions that fulfill the BLAST result conditions, with at least one domain from the InterPro database and with consensus hit description corroborated by the protein domains identified with InterProScan. This class was termed the « similar to *function* » protein and included well conserved proteins with defined functions among several organisms. All parameter values used in this section were optimized values based on comparison between results of automated *in silico* and « manual » *in silico* functional annotation.

#### Identification and annotation of genes encoding SSPs and secondary metabolite biosynthetic genes

A protein was classified as a SSP (i) if a signal peptide was predicted by both Neural-Network and Hidden Markov Model methods of SignalP 3.0 [83], (ii) if TargetP 1.1 [84] predicted the protein being in the secretion pathway, (iii) if TMHMM 2.0 [85] detected none or one transmembrane domain if this latter is at least at 30% included in the signal peptide, (iv) and if its length was ≤ 300 amino acids. SSP were identified by merging of two sets of predicted proteins. The first set resulted from a step of gene annotation as described above. After this step, the validated gene models were masked on the genomic sequences and another round of gene annotation was carried out. From this second gene model set, the ones encoding SSPs were then analysed.

Genes encoding key enzymes in fungal secondary metabolism were sought. Non-ribosomal peptide synthases (NRPS) and polyketide synthase (PKS) were identified by searching both the predicted proteins and genome assemblies of each species with previously characterised NPS and PKS proteins from Lmb v23.1.3 [9]. Both BLASTp and tBLASTn algorithms were used. Any match with greater than 35% sequence similarity to the previously identified proteins was compared by BLAST against the NCBI database to detect indicative domains, as well as best matches in other species.

#### Analysis of gene conservation

The 57,964 proteins predicted in the *Leptosphaeria* species complex were grouped using orthoMCL (with default parameters). OrthoMCL created 10,916 families that contained 48,013 sequences. In order to obtain 1:1 orthologous relationships between the sequences, families made of proteins from only one taxon or containing a number of protein larger than that of taxa, were excluded. After this screening step, 10,131 families containing 43,437 sequences (74.9% of the species complex proteome) remained.

The presence of proteins that were not included within the 10,131 families, was investigated in genomes of the other members of the species complex using different programs of BLAST with an e-value cut-off of 1e^−5^ and a request for Best Hits only: (i) BLASTn; (ii) tBLASTn on the 6 frame-translated genome sequences; (iii) BLASTp on the ungrouped proteins of the other members. All BLAST hits with coverage lower than 50% of the query sequence were considered as negative. According to the different combination of results, each protein was classified as: (i) species-specific, *i.e*. sequences that were not detected at the nucleotide level in the other assemblies or were pseudogenized; (ii) specific of a few members of the species complex, *i.e*. present in at least another member of the species complex, which can or not correspond to non-predicted genes in the other assemblies; (iii) unresolved, corresponding to sequences for we could not decide presence or absence status. Pseudogenes were hypothetized to occur when BLASTn and tBLASTn, but not BLASTp, provided significant results on other predicted proteins. To ascertain the reality of pseudogenes, the tBLASTn alignment was analyzed and the subject sequence was investigated for occurrence of stop codons or mutations in the start codon.

#### Annotation of transposable elements

Transposable Elements were identified and annotated using the REPET pipeline [86]. The TEdenovo pipeline detects repeat copies, clusters them into families and generates a consensus sequence for each family. Then these sequences are classified (TEclassifier.py) using tBLASTx and BLASTx against the Repbase Update database [87] and by identification of structural features such as long terminal repeats (LTRs) or terminal inverted repeats (TIRs), but due to the difficulties for Newbler to assemble correctly repeated regions, the majority of the consensus were not categorized into known TE families [88, 89]. Thus, manual annotation was necessary. Consensus sequences were clustered based on homologies research by BLAST, then aligned with ClustalX2 [90] and a new consensus was created. These steps were repeated until there were no more alignments by BLAST between the sequences, then consensuses were submitted to TEclassifier.py from the TEdenovo pipeline. The sequences were also translated into the six frames using Transeq from the EMBOSS package [91] in order to carry out a protein domain research on the Conserved Domain Database (CDD) [92] using RPS-BLAST. TE families of each strain were classified and named according to Wicker *et al*. [88]. These families constitute the TE repertoire of the *L. maculans* – *L. biglobosa* species complex which was used afterwards to reannotate each genome of the complex and to retrieve similar sequences among genomes of other Ascomycete species, mined on the JGI MycoCosm portal (http://genome.jgi.doe.gov/programs/fungi/index.jsf). For this latter purpose, the most GC-rich copies (*i.e*. the ones with the least degrees of RIP mutation) of the different TE families were used to search for homologous sequences in the *Ascomycota*. RIP analysis was performed automatically on multiple alignment of sequences of each TE family using the RIPCAL software [93].

#### Setting up the synteny browser

A GBrowse-based synteny browser, GBrowse-syn [94], was set up to display the synteny between genomes of the *Leptosphaeria* complex. Genome assemblies were aligned with Mercator and MAVID softwares [95]. Using Blat, Mercator aligned the CDS of all assemblies, providing constraints for genomic alignments with MAVID. For the *L. maculans* isolates, Mercator identified at least 94% of CDS as anchors for alignments (Lmb v23.1.3: 95%, Lmb WA74: 95%, Lml IBCN84: 94.3%), while it was lowered to 88% for *L. biglobosa* isolates (Lbt IBCN65: 89.5%, Lbb B3.5: 87.3%). The Lbc isolate was not included due to extensive genome fragmentation. Where the number of aligned CDS was consistent for *L. maculans* isolates, the orthology map inferred from anchors revealed that the orthologous segments covered 64% and 66% of WA74 and v23.1.3 genomes, respectively. This seemingly low coverage is due to the importance of repeated region in the genomes of Lmb isolates. The other genomes were covered at 86.5%, 89.5% and 87.3% for IBCN84, IBCN65 and B3.5, respectively. Genomic alignments were performed with MAVID and loaded with genome annotations in MySQL databases. GBrowse-syn was configured to display the blocks of synteny (http://urgi.versailles.inra.fr/gb2/gbrowse_syn/leptosphaeria_synteny/).

### Phylogeny and estimates of divergence time

An aggregative hierarchical clustering procedure was performed on all versus all BLASTp results from protein sequences of 80 annotated genomes at NCBI (Additional file 25: Table S13). This procedure is similar to that used to generate clusters in the Entrez Protein Clusters database at NCBI (ProtClustDB). The following filters were applied: cluster members were required to have compositional BLAST hits covering at least 70% of each protein length and a pairwise score between cluster members was required to be at least 20% of the largest of the self-scores. Clusters were selected that contained one protein per taxon with at least 75 taxa present. This represented a broad sampling of *Ascomycota*, *Basidiomycota* and *Chytridiomycota* orthologs. Protein sequences from these clusters were subsequently used as queries in BLASTp searches in order to extract orthologs from annotated dothideomycetes genomes at JGI (http://genome.jgi.doe.gov/programs/fungi/index.jsf; Additional file 26: Table S14) and those generated for this study. Individual protein alignments were used to generate phylogenetic trees in FastTree [96]. The phylogenies were then manually inspected for contradictory placement of taxa resulting from paralogs, or poor annotations. Taxa were judged to be contradictory when their placement above order level, in 70% bootstrap resamplings, contradicted accepted phylogenetic placements from recent broad analyses [25, 27]. The first selection of single copy clusters yielded 28 candidates. After examining the phylogenetic placements a further set of 9 proteins were removed. This series of selections yielded a final set of 19 aligned proteins. These alignments were then edited with Gblocks v.0.91b [97] with the following parameters b3 = 8 b4 = 5 b5 = h. The final concatenated alignment consisted of the following 19 proteins: Cct3p, Chc1p, Frs2p, Hsp60p, Imp3p, Kre33p, Lys1p, Pol3p, Pro2p, Pup1p, Ret1p, Rpo21p, Rpt2p, Rpt5p, Rrb1p, Rvb1p, Rvb2p, Sec26p and Tcp1p (following *Saccharomyces cerevisiae* nomenclature). The fungal taxa were trimmed to 51 in order to allow a relatively small and focused data set for analysis. This resulted in a data matrix of 11694 amino acids (aa) with 3.31% consisting of gaps and completely undetermined characters.

The data set was analysed using Bayesian relaxed molecular clock approaches in BEAST v1.7.3. An xml file was prepared with the aid of BEAUTi v.1.7.3 [98]. This included a starting tree generated in RAxML version 7.3.1 and calibrated with r8s [99]. The tree as well as details on tree analysis were deposited with the protein alignments used at TreeBASE (submission ID 15756). The same phylogeny was also used to determine which nodes could be constrained. Bayesian relaxed uncorrelated lognormal clock analyses with a birth-death tree prior were specified under the WAG substitution model (Gamma + invariant sites). Secondary time calibrations were used from recent publications: a normal distributed prior of 662 MYA was set for the root of the tree with uniform prior minimums of 417 MYA and 402 MYA for *Ascomycota* and *Basidiomycota* respectively following [100]. Maximum dates were set to 644 and 664. Similar restrictions were placed on *Dothideomycetes* with a minimum age of 284 and a maximum of 394 following Gueidan *et al*. [101]. Five independent BEAST MCMC chains were run for 15 million generations sampling data every 1000th generation using the XSEDE computing infrastructure through the CIPRES Science Gateway webportal [102]. The resulting log files were combined using LogCombiner v1.7.3 and inspected with Tracer v1.5 to confirm the estimated sample sizes. The runs converged to stable likelihood values independently and the first 1 500 trees were discarded. The remaining 13 500 ultrametric trees from each run were combined and analyzed using TreeAnnotator v1.7.3 to estimate the 95% highest posterior densities (HPD). The consensus chronogram including the 95% HPD and the mean age estimates were visualised in Figtree v1.3.1 and certain clades were collapsed for clarity in Figure 1. The full figure is available as Additional file 2: Figure S2.

### Gene expression data

Expression data based on oligoarrays for isolate Lmb v.23.1.3 previously obtained [9] and deposited in the gene Expression Omnibus under accession code GSE27152 were used. In addition, whole-genome expression arrays of Lbb isolate B3.5 were obtained and analysed. They were manufactured by NimbleGen Systems Limited (Madison, WI) and contained five independent, non-identical, 60-mer probes per gene model. A total of 12,457 gene models were analysed with 2,008 random 60-mer as well as control probes and labelling controls. Total RNA was extracted from mycelium grown during one week in Fries liquid medium and from oilseed rape leaves after 3, 7 and 14 days post-infection using TRIzol reagent (Invitrogen) according to the manufacturer’s protocol. Total RNA was treated with DNase I RNase-Free (New England Biolabs). Total RNA preparations (three biological replicates for each sample) were amplified by PartnerChip (Evry, France) using the SMART PCR cDNA Synthesis Kit (Invitrogen) according to the manufacturer’s instructions. Single dye labeling of samples, hybridization procedures, data acquisition, background correction and normalisation were performed at the PartnerChip facilities following the standard protocol defined by NimbleGen [103, 104]. Average expression levels were calculated for each gene from the independent probes on the array and were further analyzed. Gene-normalized data were subjected to Analysis of NimbleGen Array Interface Suite [105]. ANAIS performs an ANOVA test on log-10 transformed data to identify statistically differentially expressed genes. This test uses the observed variance of gene measurements across the three replicated experiments. To deal with multiple testings, the ANOVA *p* values are further subjected to the Bonferroni correction. Transcripts with a *p* value lower than 0.05 and more than 1.5-fold change in transcript level were considered as significantly differentially expressed during *in planta* conditions (3, 7 or 14 dpi) compared to mycelium growth. To estimate the signal-to-noise threshold (signal background), ANAIS calculates the median of the intensity of all of the random probes present on the oligoarray, and provides adjustable cut-off levels relative to that value. Gene models with an expression higher than three-times the median of random probe intensities in at least two of three biological replicates were considered as transcribed.

In parallel, RNAseq analyses were performed on another Lmb isolate and on Lbc. Mycelia of Lmb IBCN18 and of Lbc J154 were harvested on Miracloth after 7 days growth in oilseed rape liquid medium. Infected cotyledonary tissue (0.5 mm diameter) was harvested from 32 *B. napus* cv. Westar seedlings 7 days post inoculation (dpi) [106]. RNA was extracted using Trizol reagent (Life technologies) and subsequently DNaseI-treated (Promega). Two biological replicates of RNA, with an RNA integrity number (RIN) > 6, were sequenced with Illumina TruSeq version 3 chemistry on an Illumina HiSeq2000 sequencer at the Australian Genome Research Facility (AGRF). *In vitro* derived RNA was sequenced with 100 bp paired-end reads in order to aid gene annotation, while *in planta* derived RNA was sequenced with 100 bp single-end reads. A total of 15.5 Gb sequence was generated from two i*n vitro* libraries (7.75 Gb per sample), and 72 Gb sequence was generated from 12 *in planta* libraries (6 Gb per sample). Reads were trimmed to a minimum phred score of 20 using Nesoni sequence software and remaining Illumina Tru-seq adaptor sequences were removed. Trimmed reads were aligned to reference genome sequences with Tophat v1.4.1 splice-junction mapper [107]. Reference genomes were *L. maculans* ‘brassicae’ v23.1.3 [9] *L. biglobosa* ‘canadensis’ isolate J154 (this study). Aligned reads were quantified using Cufflinks v1.0.3 transcript assembly and quantification software [108].

Gene models for Lbc isolate J154 were validated with the RNA-seq sequence data. Of the 11068 gene models, 8521 had >10 x coverage, 9979 had >1x coverage and 530 did not have a detectable transcript. Of the 11068 gene models, 8948 had a FPKM (fragments per kb of exon per million mapped reads) value >1; this value is an indication of significant expression.

#### Effect of proximity of repetitive sequences on in planta expression of genes

A total of 7561 orthologous pairs between isolates Lmb IBCN18 and Lbc J154 were identified and RNA-seq gene expression values (FPKM) for each gene in two conditions, *in planta* (infected *B. napus* cv Westar at 7 dpi), and *in vitro* (oilseed rape medium at 7 dpi) were determined. Genes with undetectable expression (no assignable FPKM) were excluded, leaving 7415 ortholog pairs. Quantile Normalisation was applied to the four gene expression datasets so that they could be directly compared. The repeat category for each ortholog pair was determined: the RepeatMasker output based on TE families described above defined the repeat content of each genome, and all repetitive sequences were included in the analysis. For each gene considered, either another gene or a repetitive sequence was adjacent; if a gene was adjacent to the end of a scaffold, it was categorised as “repetitive sequence adjacent”. This led to 16 categories based on the presence/absence of a repetitive sequence adjacent to promoter and terminator for orthologs in Lmb and Lbc. However, four categories based on the promoter region were analysed. An expression ratio was calculated from the log2 (*in planta* expression /i*n vitro* expression) for each gene. A positive value means the gene is more highly expressed *in planta*; a negative value means the gene is more highly expressed *in vitro* culture.

### Ethics

Not applicable; this research is essentially a bioinformatics-based one and did not involve research on human, animals or plants.

### Availability of supporting data

Tree and alignment have been submitted to TreeBase as matrix S15756. They are available at http://purl.org/phylo/treebase/phylows/study/TB2:S15756.

*Leptosphaeria biglobosa* ‘canadensis’ genome information and RNAseq data have been deposited to genbank linked to NCBI Bioproject ID "PRJNA230885”.

Assembly for all other genomes have been deposited to EBI under accession numbers FO905058 - FO907085.

Sequences of Transposable Elements of the species complex are downloadable from https://urgi.versailles.inra.fr/Species/Leptosphaeria/Sequences-Databases/Download.

## Electronic supplementary material

Additional file 1: Figure S1: Representative electrokaryotypes and presence of transposable elements in the genomes of isolates of the *L. maculans*-*L. biglobosa* species complex. (a) Electrokaryotypes were separated by Contour-clamped Homogeneous Electric Field (CHEF) electrophoresis. (b) Southern blotting using one probe derived from the retrotransposon RLG_*Rolly*. Identity of the isolates is as follows *L. maculans* ‘brassicae’: lane 1, v23.1.3; lane 2, v29; lane 3, IBCN18; *L. maculans* ‘lepidii’: lane 4, IBCN84; lane 5, Lepi-1; lane 6, Lepi-2; *L. biglobosa* ‘brassicae’: lane 7, IBCN10; lane 8, IBCN93; *L. biglobosa* ‘canadensis’: lane 9, IBCN62; lane 10, IBCN82; *L. biglobosa* ‘australensis’: lane 12, IBCN30; lane 13, IBCN91; *L. biglobosa* ‘thlaspii’: lane 13, IBCN65; lane 14, IBCN64; lane 15, molecular weight marker *H. wingei* chromosomes. Names of isolates in bold are those sequenced here or the reference isolate previously sequenced [9]. (PNG 351 KB)

Additional file 2: Figure S2: Expanded chronogram of major classes in *Ascomycota*, with a focus on *Dothideomycetes*, produced with BEAST from a data set of 19 truncated proteins. Numbers at nodes indicate mean node ages in millions of years and light green bars indicate their 95% highest posterior density intervals. A broken line indicates uncertain phylogenetic placement. (PNG 571 KB)

Additional file 3: Figure S5: Genomic DNA of isolates of *Leptosphaeria* species digested with restriction enzymes *Bam*HI (RLC_*Pholy*) or *Hind*III (RLG_*Rolly*) and hybridised with probes of transposable elements abundant in *L. maculans* ‘brassicae’. (a, b) Probed with RLG_*Rolly*; RLC_*Pholy*, respectively. (c, d) Ethidium bromide stained gel of (a, b), respectively. None of the probes hybridised to DNA of *L. biglobosa* ‘canadensis’ or with another Australian member of the species complex (not included in the present study), *L. biglobosa* ‘occiaustraliensis’. (PNG 124 KB)

Additional file 4: Table S1: Characteristics of selected genomes of *Dothideomycetes*. (XLSX 13 KB)

Additional file 5: Table S2: Gene model statistics for the *Leptosphaeria* spp. genomes. (XLSX 18 KB)

Additional file 6: Table S3: Chromosomal assignment and correspondance between *L. maculans* ‘brassicae’ and *L. maculans* ‘lepidii’. (XLSX 14 KB)

Additional file 7: Figure S3: Whole genome DNA comparison of *Leptosphaeria maculans* ‘brassicae’ isolate v23.1.3 to progressively more distantly related members of the species complex. (a) comparison between two *Leptosphaeria maculans* ‘brassicae’ isolates, v23.1.3 and WA74 showing lack of detectable chromosomal reorganisations; (b) comparison with *L. maculans* ‘lepidii’ showing extensive macrosynteny with only limited number of intrachromosomal inversions; (c) comparison to *Leptosphaeria biglobosa* ‘brassicae’ and (d) comparison to *Leptosphaeria biglobosa* ‘thlaspii’ showing many intrachromosomal inversions but no detectable large scale translocations. (PNG 412 KB)

Additional file 8: Figure S6: Circos representation of chromosome-by-chromosome genome conservation between *L. maculans* ‘brassicae’ (right part of the diagramme) and *L. maculans* ‘lepidii’ (left part). For *L. maculans* ‘brassicae’, each chromosome is represented in a different colour and black blocks within coloured bars represent AT-rich genome blocks enriched in transposable elements. (PNG 2 MB)

Additional file 9: Table S4: Location and distance from TEs of the 30 intrachromosomal inversions in the chromosomes of *L. maculans* ‘brassicae’. (XLSX 18 KB)

Additional file 10: Table S5: Characteristics of Class I (Retrotransposons) Transposable Elements identified in the *Leptosphaeria maculans*-*L. biglobosa* species complex. (XLSX 17 KB)

Additional file 11: Table S6: Characteristics of Class II (DNA transposons) Transposable Elements identified in the *Leptosphaeria maculans*-*L. biglobosa* species complex. (XLSX 18 KB)

Additional file 12: **Supplementary Data 1.** Unclassified repeated elements and their occurrence in species of the *L. maculans*-*L. biglobosa* species complex. (XLSX 10 KB)

Additional file 13: **Supplementary Data 2.** Occurrence and conservation of annotated families of transposable elements in the *Pleosporales*. (XLSX 18 KB)

Additional file 14: Figure S4: Transposable Elements dynamics in the *Dothideomycetes* lineage. Red stars indicate species genomes in which TE sequences identified in the *L. maculans*-*L. biglobosa* species complex are present. (PNG 817 KB)

Additional file 15: Table S7: Insertion polymorphism of Transposable Elements between the two isolates of *L. maculans* ‘brassicae’. (XLS 9 kb). (XLS 10 KB)

Additional file 16: Table S8: Predicted gene conservation between the genomes of isolates of the *Leptosphaeria maculans*-*Leptosphaeria biglobosa* species complex. (XLSX 9 KB)

Additional file 17: Figure S7: Conservation of cysteine spacing in a series of orthologs of avirulence proteins of *L. maculans* ‘brassicae’. (a) multiple alignment of orthologs of AvrLm4-7, (b) multiple alignment of orthologs of AvrLm6. (PNG 1 MB)

Additional file 18: Table S9: Conservation of genes encoding avirulence effector in members of the *L. maculans*-*L. biglobosa* species complex and other fungal species. (XLSX 17 KB)

Additional file 19: Table S10: Non-Ribosomal Peptide Synthases (NPS) genes of the *L. maculans*-*L. biglobosa* species complex. (XLSX 16 KB)

Additional file 20: **Supplementary Data 3.** Top 10 Blast hit for the secondary metabolite genes (PKS and NPS) found in members of the *L. maculans*-*L. biglobosa* species complex. (XLSX 23 KB)

Additional file 21: Table S11: Polyketide Synthases (PKS) genes of the *L. maculans*-*L. biglobosa* species complex. (XLSX 17 KB)

Additional file 22: Table S12: The PKS21 gene cluster of *Leptosphaeria biglobosa* ‘brassicae’ and homologs in the dermatophyte *Arthroderma otae*. (XLSX 10 KB)

Additional file 23: **Supplementary Data 4.** Top 100 genes over-expressed at 7dpi in species adapted to oilseed rape, Lmb, Lbb and Lbc. (XLSX 18 KB)

Additional file 24: Figure S8: Typical symptoms caused by *Leptosphaeria maculans* (a) and *Leptosphaeria biglobosa* (b) on cotyledons of oilseed rape following infection in controlled environment. In (a) two different isolates were inoculated, one causing the typical susceptibility symptom (left) and one expressing an avirulence effector recognised by the Effector Triggered Immunity plant machinery (right) resulting in a typical hypersensistive response to infection. In (b), susceptibility symptoms differ from that in (a) by the occurrence of numerous dark necrotic spots and development of chlorosis never observed in (a). (PNG 3 MB)

Additional file 25: Table S13: List of taxa which predicted protein annotation (available in 2012) were used in hierarchical clustering procedure. (XLSX 10 KB)

Additional file 26: Table S14: List of dothideomycetes taxa with annotated genomes (available in 2012) from which orthologs were identified. (DOCX 11 KB)
