# Supplementary figures and images for: Transposable element-assisted evolution and adaptation to host plant within the Leptosphaeria maculans-Leptosphaeria biglobosa species complex of fungal pathogens
Source: BMC Genomics. 2014 Oct 12;15:891. doi: 10.1186/1471-2164-15-891 (PMC4210507; doi:10.1186/1471-2164-15-891)

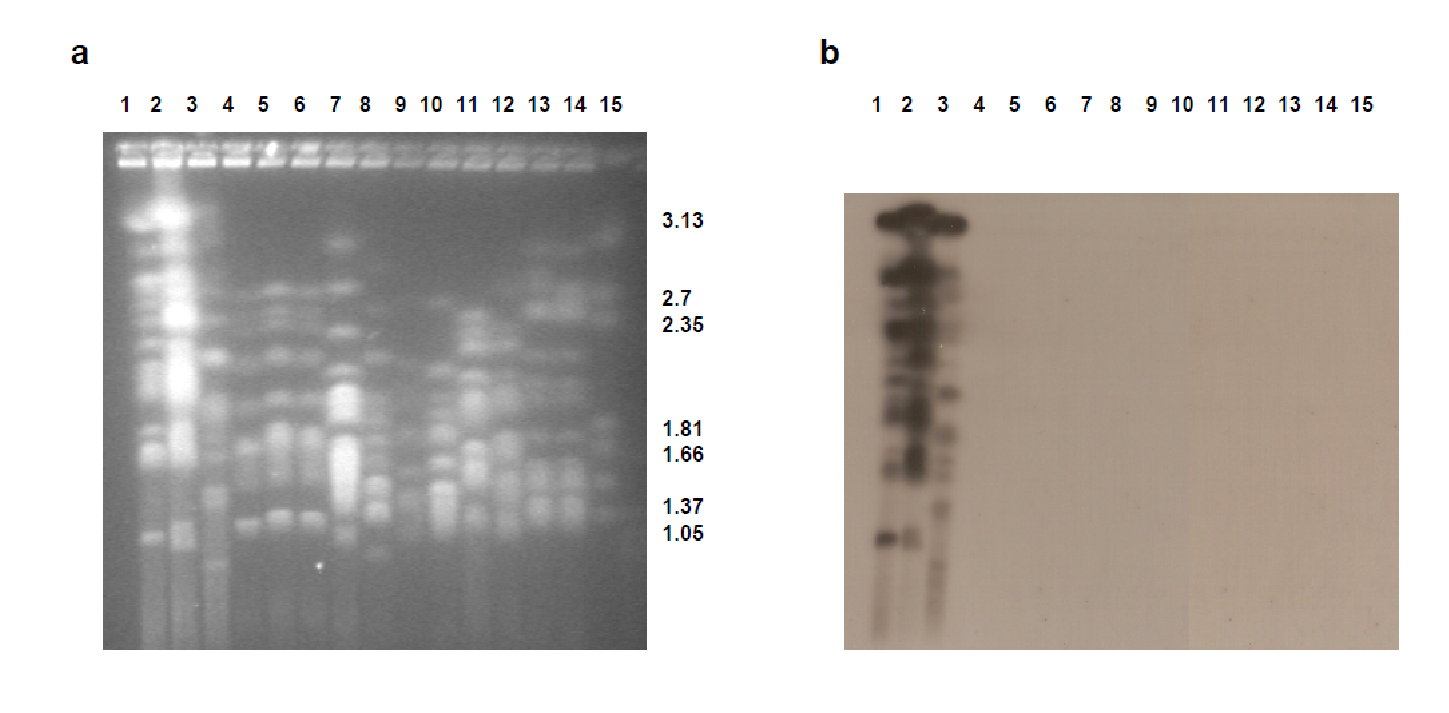

Supplement: Supplementary file 1 — Additional file 1: Figure S1: Representative electrokaryotypes and presence of transposable elements in the genomes of isolates of the L. maculans-L. biglobosa species complex. (a) Electrokaryotypes were separated by Contour-clamped Homogeneous Electric Field (CHEF) electrophoresis. (b) Southern blotting using one probe derived from the retrotransposon RLG_Rolly. Identity of the isolates is as follows L. maculans ‘brassicae’: lane 1, v23.1.3; lane 2, v29; lane 3, IBCN18; L. maculans ‘lepidii’: lane 4, IBCN84; lane 5, Lepi-1; lane 6, Lepi-2; L. biglobosa ‘brassicae’: lane 7, IBCN10; lane 8, IBCN93; L. biglobosa ‘canadensis’: lane 9, IBCN62; lane 10, IBCN82; L. biglobosa ‘australensis’: lane 12, IBCN30; lane 13, IBCN91; L. biglobosa ‘thlaspii’: lane 13, IBCN65; lane 14, IBCN64; lane 15, molecular weight marker H. wingei chromosomes. Names of isolates in bold are those sequenced here or the reference isolate previously sequenced [9]. (PNG 351 KB) [file 12864_2014_6595_MOESM1_ESM.png]

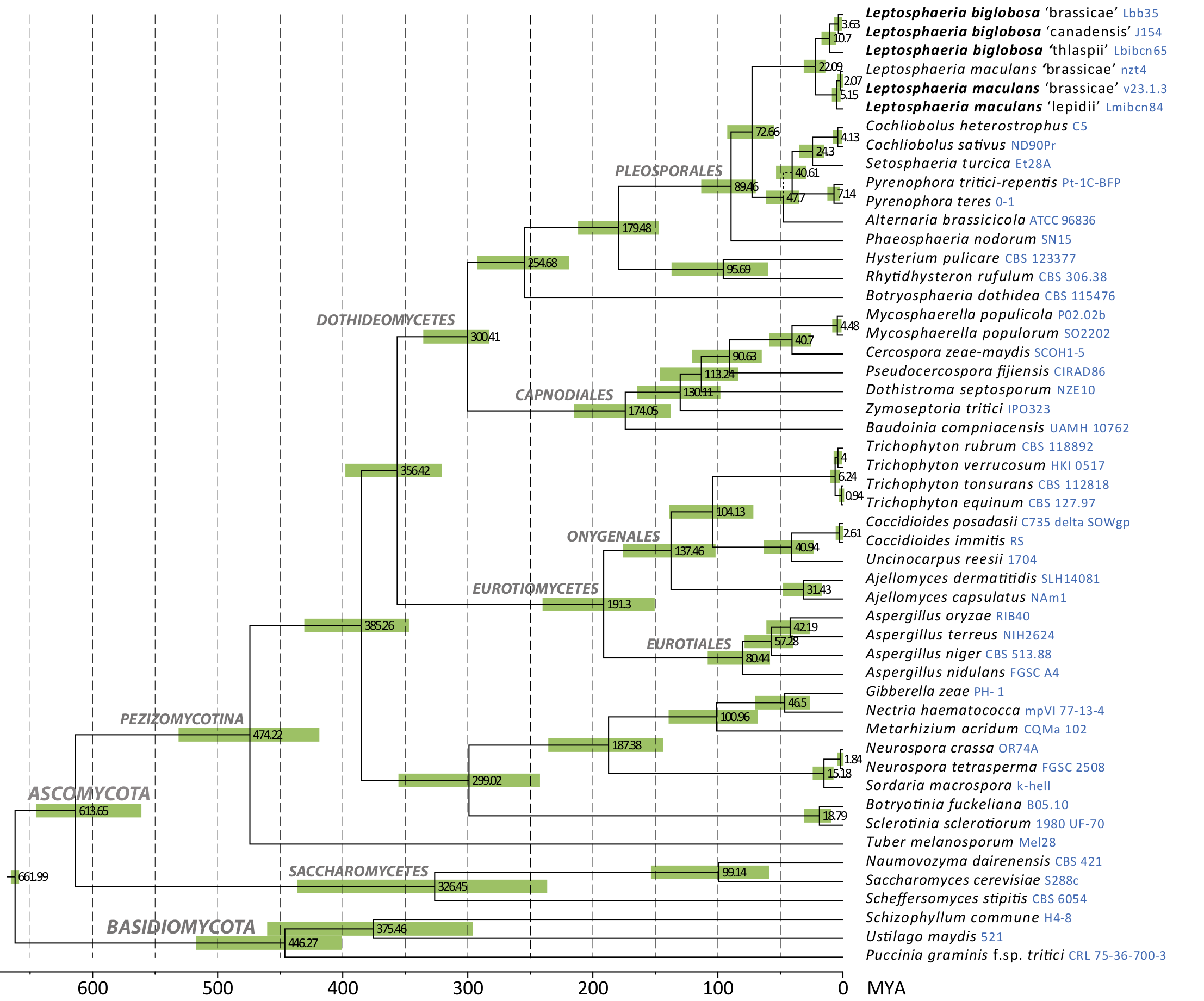

Supplement: Supplementary file 2 — Additional file 2: Figure S2: Expanded chronogram of major classes in Ascomycota, with a focus on Dothideomycetes, produced with BEAST from a data set of 19 truncated proteins. Numbers at nodes indicate mean node ages in millions of years and light green bars indicate their 95% highest posterior density intervals. A broken line indicates uncertain phylogenetic placement. (PNG 571 KB) [file 12864_2014_6595_MOESM2_ESM.png]

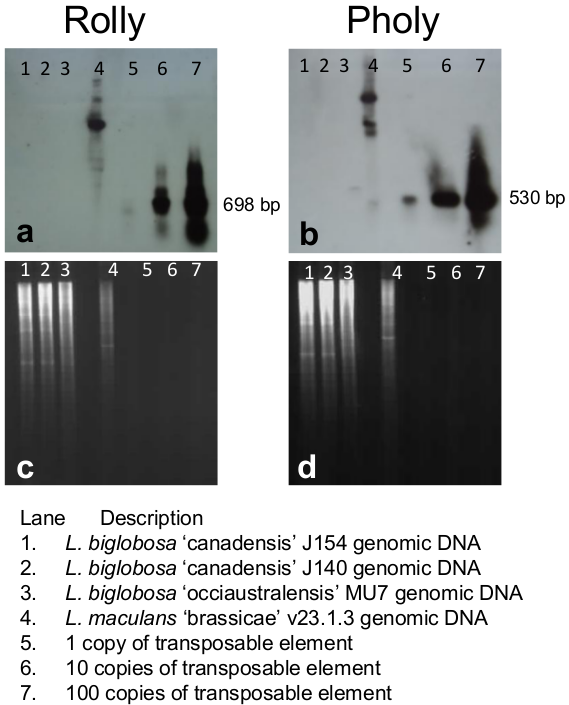

Supplement: Supplementary file 3 — Additional file 3: Figure S5: Genomic DNA of isolates of Leptosphaeria species digested with restriction enzymes BamHI (RLC_Pholy) or HindIII (RLG_Rolly) and hybridised with probes of transposable elements abundant in L. maculans ‘brassicae’. (a, b) Probed with RLG_Rolly; RLC_Pholy, respectively. (c, d) Ethidium bromide stained gel of (a, b), respectively. None of the probes hybridised to DNA of L. biglobosa ‘canadensis’ or with another Australian member of the species complex (not included in the present study), L. biglobosa ‘occiaustraliensis’. (PNG 124 KB) [file 12864_2014_6595_MOESM3_ESM.png]

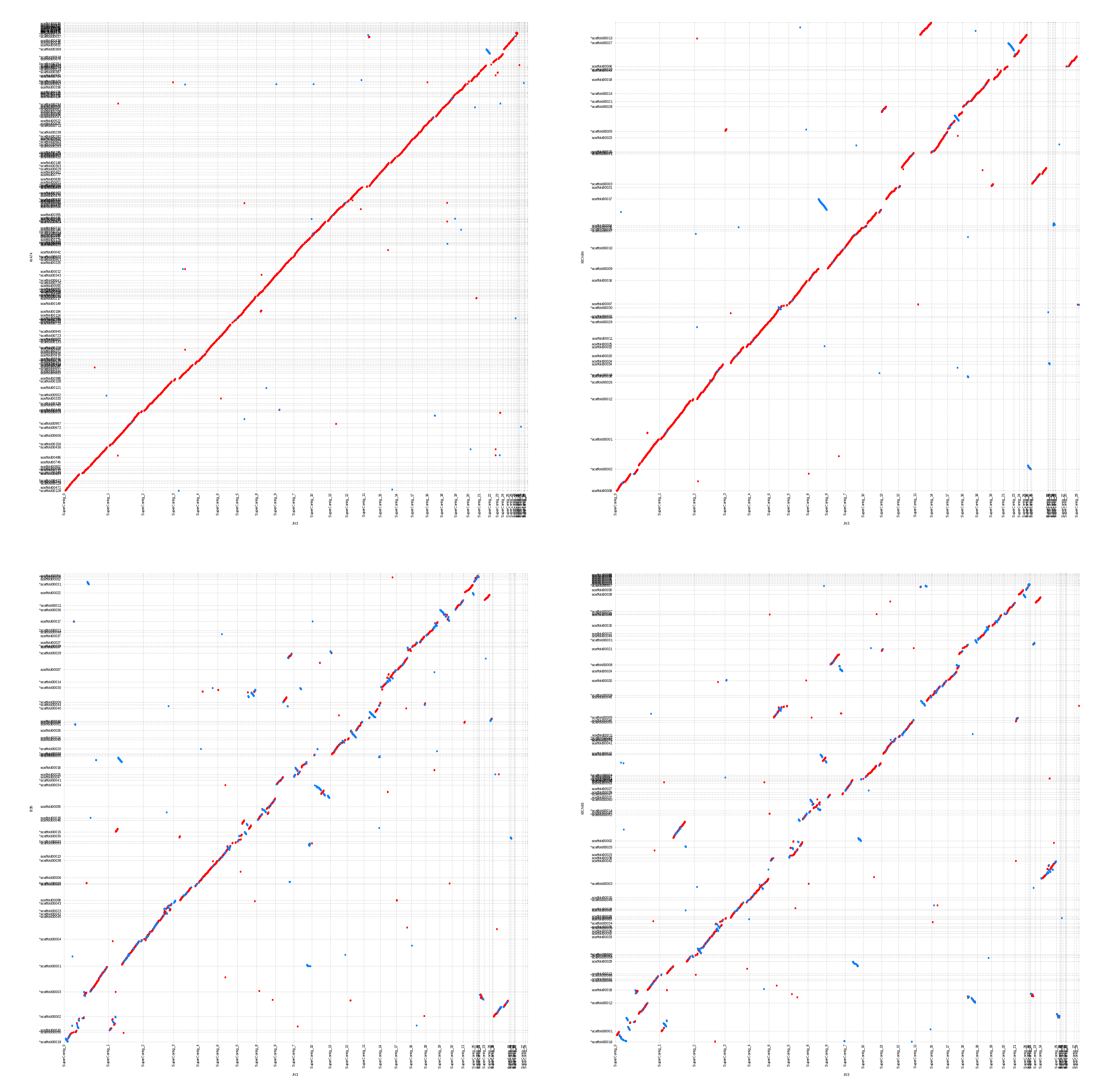

Supplement: Supplementary file 7 — Additional file 7: Figure S3: Whole genome DNA comparison of Leptosphaeria maculans ‘brassicae’ isolate v23.1.3 to progressively more distantly related members of the species complex. (a) comparison between two Leptosphaeria maculans ‘brassicae’ isolates, v23.1.3 and WA74 showing lack of detectable chromosomal reorganisations; (b) comparison with L. maculans ‘lepidii’ showing extensive macrosynteny with only limited number of intrachromosomal inversions; (c) comparison to Leptosphaeria biglobosa ‘brassicae’ and (d) comparison to Leptosphaeria biglobosa ‘thlaspii’ showing many intrachromosomal inversions but no detectable large scale translocations. (PNG 412 KB) [file 12864_2014_6595_MOESM7_ESM.png]

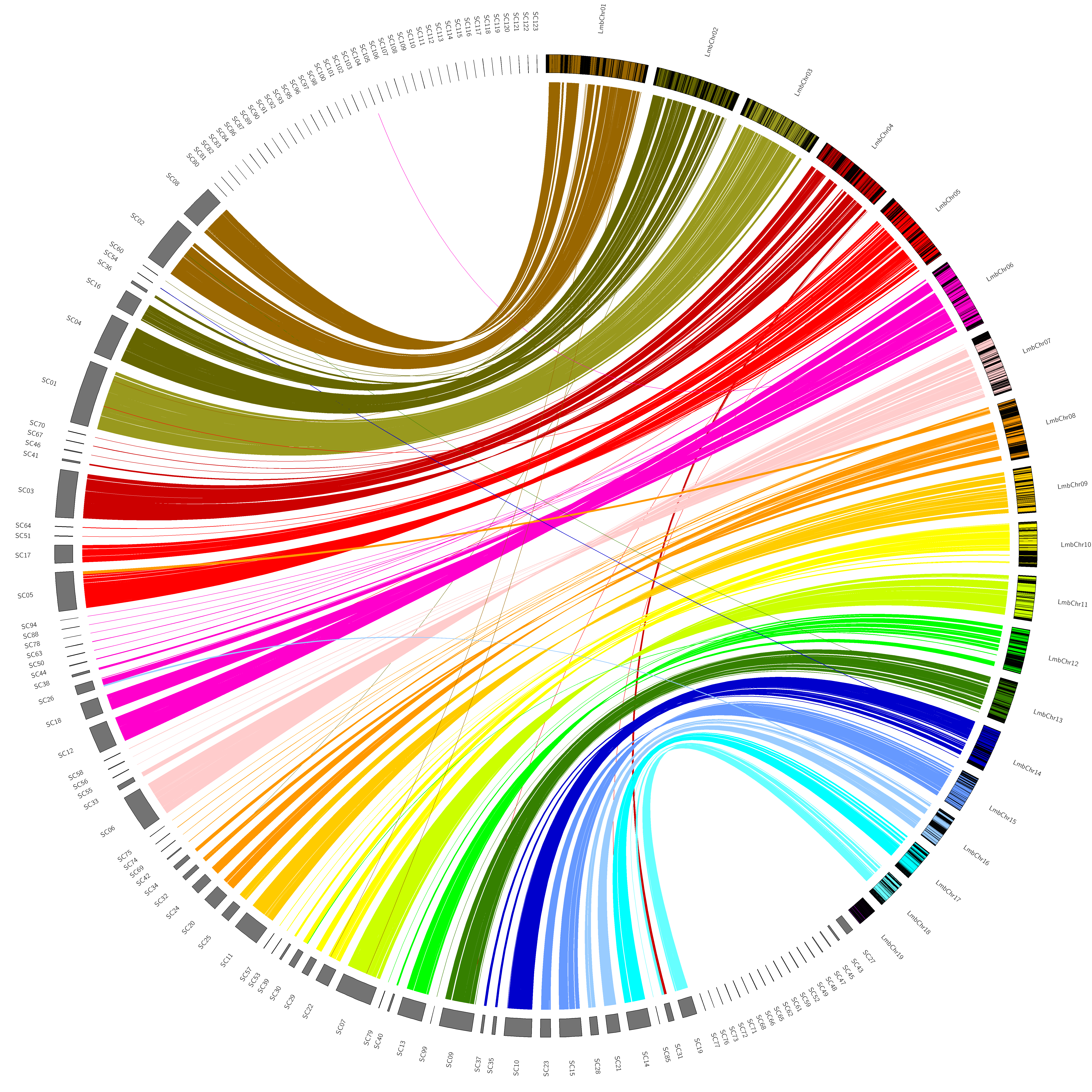

Supplement: Supplementary file 8 — Additional file 8: Figure S6: Circos representation of chromosome-by-chromosome genome conservation between L. maculans ‘brassicae’ (right part of the diagramme) and L. maculans ‘lepidii’ (left part). For L. maculans ‘brassicae’, each chromosome is represented in a different colour and black blocks within coloured bars represent AT-rich genome blocks enriched in transposable elements. (PNG 2 MB) [file 12864_2014_6595_MOESM8_ESM.png]

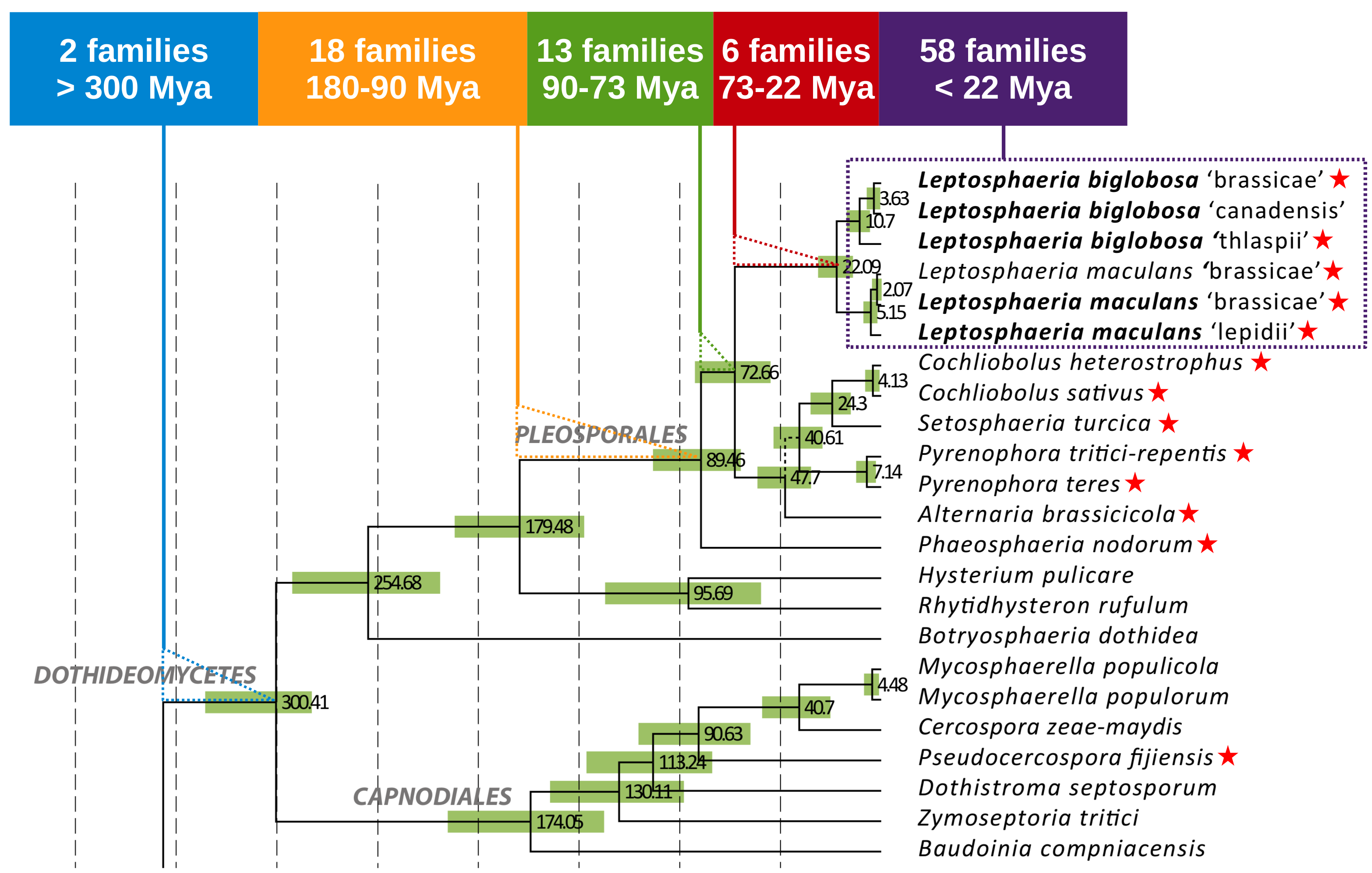

Supplement: Supplementary file 14 — Additional file 14: Figure S4: Transposable Elements dynamics in the Dothideomycetes lineage. Red stars indicate species genomes in which TE sequences identified in the L. maculans-L. biglobosa species complex are present. (PNG 817 KB) [file 12864_2014_6595_MOESM14_ESM.png]

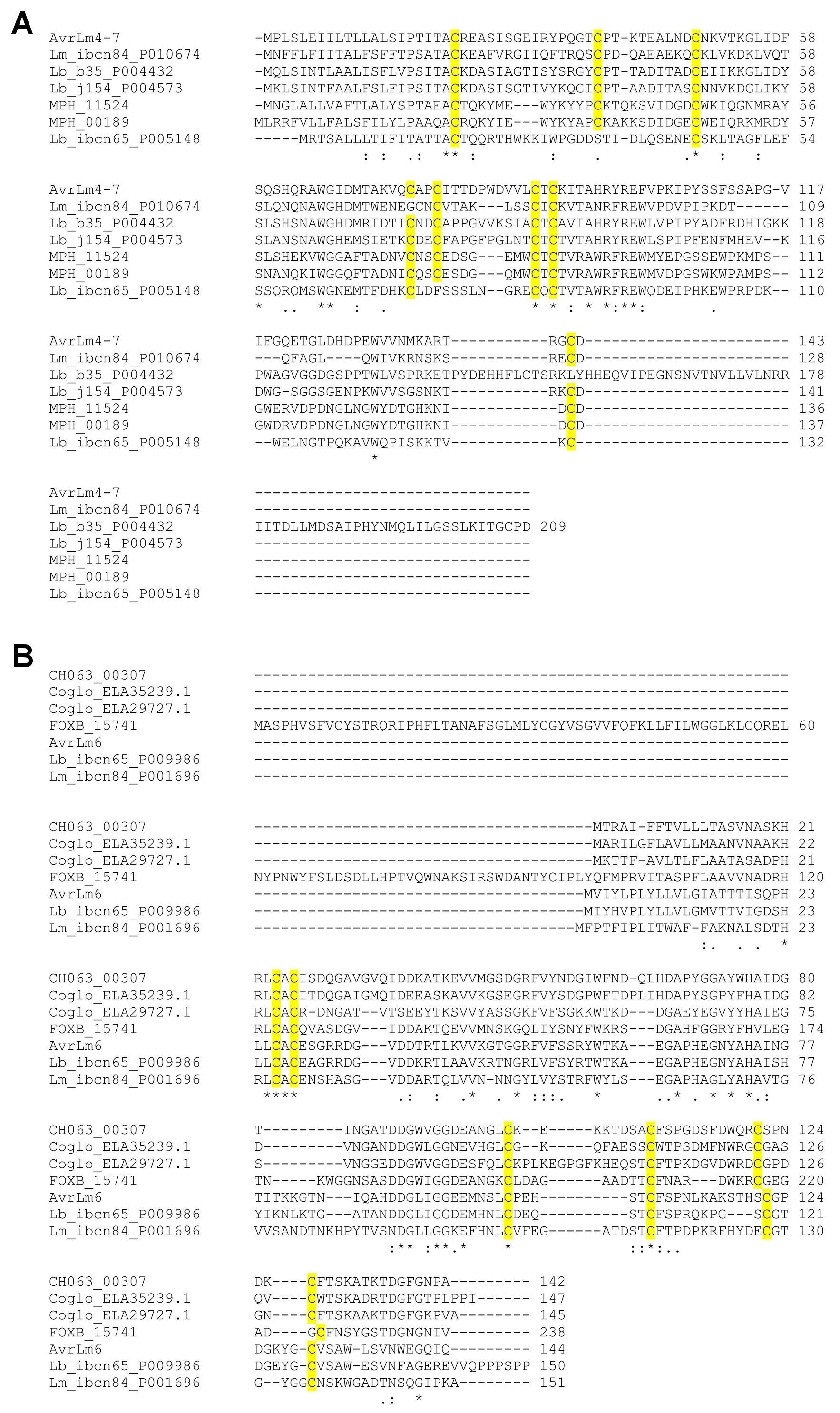

Supplement: Supplementary file 17 — Additional file 17: Figure S7: Conservation of cysteine spacing in a series of orthologs of avirulence proteins of L. maculans ‘brassicae’. (a) multiple alignment of orthologs of AvrLm4-7, (b) multiple alignment of orthologs of AvrLm6. (PNG 1 MB) [file 12864_2014_6595_MOESM17_ESM.png]

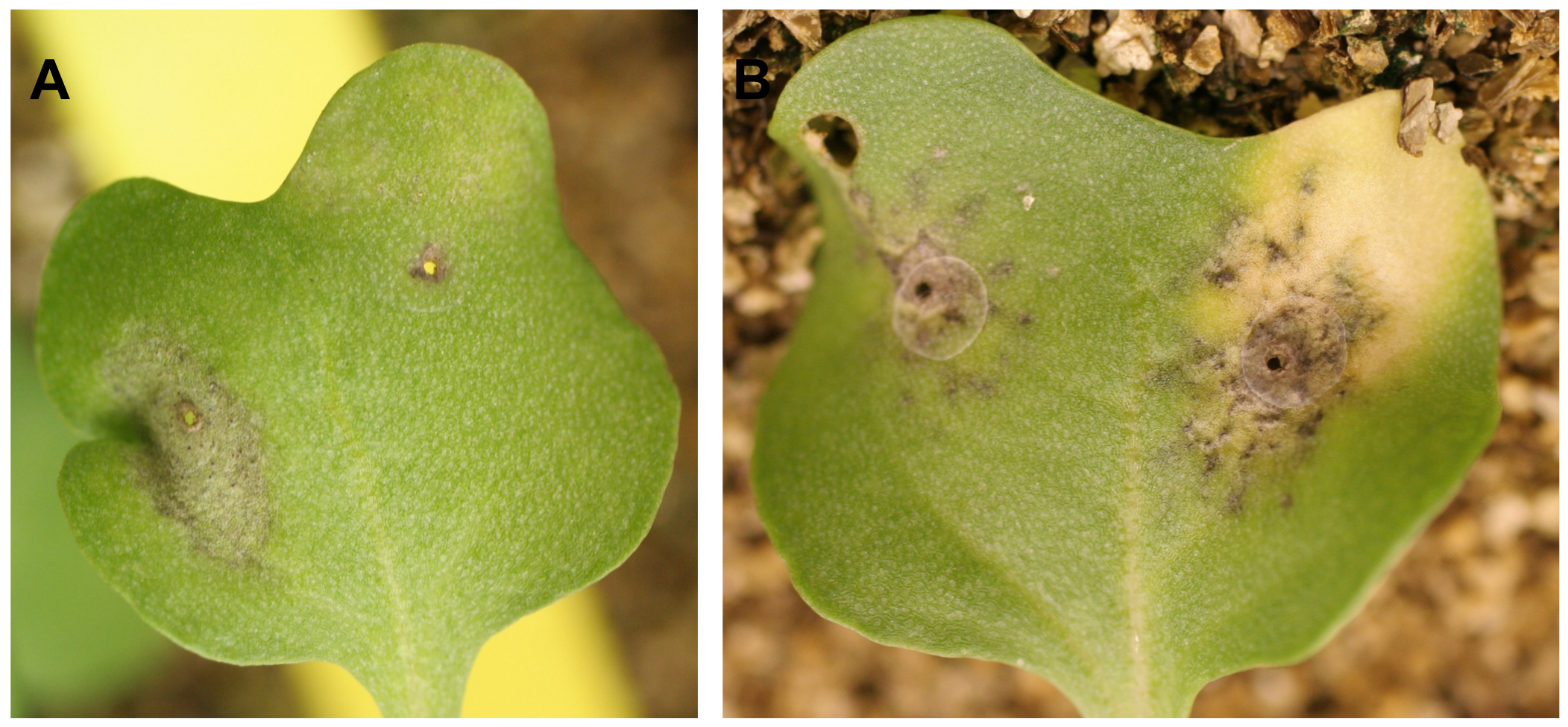

Supplement: Supplementary file 24 — Additional file 24: Figure S8: Typical symptoms caused by Leptosphaeria maculans (a) and Leptosphaeria biglobosa (b) on cotyledons of oilseed rape following infection in controlled environment. In (a) two different isolates were inoculated, one causing the typical susceptibility symptom (left) and one expressing an avirulence effector recognised by the Effector Triggered Immunity plant machinery (right) resulting in a typical hypersensistive response to infection. In (b), susceptibility symptoms differ from that in (a) by the occurrence of numerous dark necrotic spots and development of chlorosis never observed in (a). (PNG 3 MB) [file 12864_2014_6595_MOESM24_ESM.png]
