# Supplementary material for: Transposable element-assisted evolution and adaptation to host plant within the Leptosphaeria maculans-Leptosphaeria biglobosa species complex of fungal pathogens
Source: BMC Genomics. 2014 Oct 12;15:891. doi: 10.1186/1471-2164-15-891 (PMC4210507; doi:10.1186/1471-2164-15-891)
Supplement: Supplementary file 26 — Additional file 26: Table S14: List of dothideomycetes taxa with annotated genomes (available in 2012) from which orthologs were identified. (DOCX 11 KB) [file 12864_2014_6595_MOESM26_ESM.docx]

| Table S14. List of dothideomycetes taxa with annotated genomes (available in 2012) from which orthologs were identified |
| --- |
| Taxa |
| Alternaria brassicicola ATCC 96836 |
| Baudoinia compniacensis UAMH 10762 |
| Botryosphaeria dothidea CBS 115476 |
| Cercospora zeae-maydis SCOH1-5 |
| Cochliobolus heterostrophus C4 |
| Cochliobolus heterostrophus C5 |
| Cochliobolus sativus ND90Pr |
| Dothistroma septosporum NZE10 |
| Hysterium pulicare CBS 123377 |
| Mycosphaerella fijiensis CIRAD86 |
| Rhytidhysteron rufulum CBS 306.38 |
| Mycosphaerella populorum SO2202 |
| Setosphaeria turcica Et28A |
